# Integrated Single‐Cell and Spatial Transcriptomics Reveal Convergence on a Senescence‐Associated B Cell–MIF Signaling Axis in Joint Aging and Osteoarthritis Progression

**DOI:** 10.1002/advs.78543

**Published:** 2026-10-29

**Authors:** Weida Li, Yi Wang, Yongqing You, Yue Cui, Jiayun Liu, Xingbiao Ma, QiuYu Zhu, Qiang Wu, Zanjing Zhai, Zuyan Lu, Kerong Dai, Ye Sun

**Affiliations:** ^1^ Department of Orthopaedics The First Affiliated Hospital of Nanjing Medical University Jiangsu China; ^2^ Shanghai Key Laboratory of Orthopaedic Implants Department of Orthopaedic Surgery Shanghai Ninth People's Hospital Shanghai Jiao Tong University School of Medicine Shanghai China; ^3^ Clinical and Translational Research Center for 3D Printing Technology Shanghai Ninth People's Hospital Shanghai Jiao Tong University School of Medicine Shanghai China; ^4^ Department of Nephrology Nanjing Hospital of Chinese Medicine Affiliated to Nanjing University of Chinese Medicine Nanjing China

**Keywords:** B cells, cartilage degeneration, cellular senescence, macrophage migration inhibitory factor, osteoarthritis

## Abstract

Osteoarthritis (OA) involves age‐associated structural and immune changes, yet whether senescence‐associated B‐cell states contribute to cartilage degeneration remains unclear. Integrated single‐cell RNA sequencing and spatial transcriptomics of healthy, aged, and OA mouse joints identify reduced B‐cell abundance but enrichment of a low‐proliferative, inflammatory state with heightened macrophage migration inhibitory factor (MIF) signaling. Cell–cell communication and spatial ligand–receptor analyses identify enhanced predicted MIF–CD74/CD44 interactions in B‐cell‐associated regions adjacent to cartilage. Constitutive MIF deficiency attenuates senescence‐associated B‐cell changes, MMP13 induction, and cartilage damage in vivo. Joint‐derived B cells from aged and OA mice induce chondrocyte senescence, reduce COL2A1 and HMGB1, and increase FN1 and MMP13 through MIF‐dependent paracrine signaling. These effects are suppressed by MIF neutralization and restored by recombinant MIF in MIF‐deficient co‐cultures. A composite hydrogel carrying quercetin and MIF‐siRNA‐loaded PLGA microspheres for local co‐delivery. The microspheres show gradual siRNA release over 96 h in vitro. In DMM‐induced OA, the formulation reduces MIF, P16, and MMP13 signals, restores HMGB1, and preserves cartilage. These findings support a senescence‐associated B cell–MIF signaling axis associated with joint aging and chondrocyte dysfunction and provide proof of concept for a local biomaterial‐based strategy that attenuates OA‐associated changes.

## Graphical abstract

Single‐cell and spatial transcriptomics implicate senescent B cells and macrophage migration inhibitory factor (MIF) signaling in aging and osteoarthritic joints. An injectable hydrogel combining quercetin with siRNA targeting MIF attenuates cartilage degeneration in a mouse model of osteoarthritis. These findings support local targeting of B cell senescence and MIF signaling as a potential therapeutic strategy.

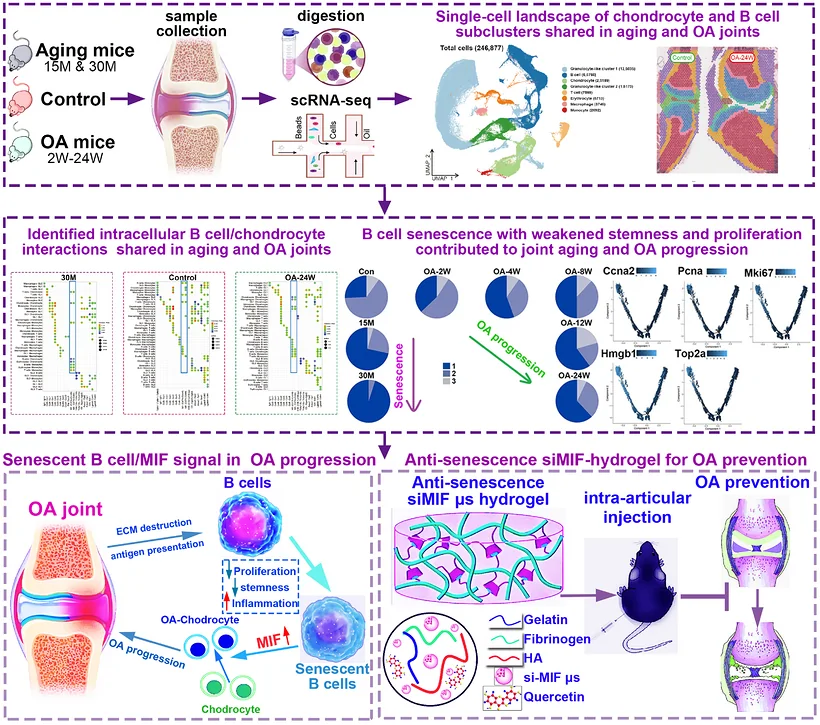

## 1 Introduction

Osteoarthritis (OA) is a chronic age‐associated joint disease characterized by progressive cartilage loss and joint dysfunction [1, 2]. More than 500 million people worldwide live with OA, making it a leading cause of disability and a growing public‐health burden [3]. Despite major advances in defining OA‐associated molecular pathways, clinically effective disease‐modifying therapies remain limited [4, 5, 6]. This therapeutic gap may partly reflect the complexity of OA, which is driven not by a single pathway but by persistent interactions between damaged structural tissues and dysregulated cell populations within the joint [7]. Stress and damage associated signals can reshape intercellular communication, sustain inflammatory and catabolic responses, recruit immune cells, and impair chondrocyte homeostasis [8, 9]. Therefore, identifying disease‐associated immune‐cell states and defining how their altered signaling activities disrupt cartilage homeostasis may uncover new pathogenic mechanisms and therapeutic targets in OA.

Immune dysregulation has been studied extensively in autoimmune and inflammatory arthritis [10, 11, 12], whereas the contribution of B cells to OA remains incompletely defined [13]. Recent studies indicate that the proliferative capacity and differentiation potential of B cells are altered in OA [14, 15, 16]. However, whether these alterations merely reflect the inflammatory joint environment or represent a senescence‐associated, pathogenic B‐cell state that actively contributes to cartilage degeneration remains unclear. Defining whether senescence‐associated B‐cell remodeling drives OA progression and joint aging may reveal new immune‐modulatory strategies for preserving cartilage homeostasis.

Macrophage migration inhibitory factor (MIF) is a pleiotropic cytokine that coordinates innate and adaptive immune responses [17, 18]. In systemic lupus erythematosus and multiple sclerosis, MIF contributes to B‐cell survival, activation, and inflammatory cytokine production [19, 20]. MIF has also been implicated in several degenerative disorders and is under investigation as a therapeutic target [21, 22]. In a mouse model of spondyloarthritis, genetic deletion of MIF alleviates disease severity [23]. Its role in OA is less clear, however, because experimental studies have reached divergent conclusions [24, 25]. These observations led us to examine whether MIF couples disease‐associated B‐cell remodeling to cartilage degeneration within the OA joint.

Single‐cell RNA sequencing and spatial transcriptomics provide complementary approaches for resolving joint‐cell heterogeneity, state transitions, intercellular communication, and anatomical context [26, 27]. Here, we integrated single‐cell profiles from healthy, naturally aged, and destabilization of the medial meniscus (DMM)‐induced OA mouse joints with spatial transcriptomic maps of control and late‐stage OA tissue. We then combined tissue‐level validation, constitutive MIF deficiency, joint‐derived B cell–chondrocyte co‐culture, MIF neutralization, and recombinant MIF add‐back to test whether MIF mediates the effects of senescence‐associated B cells on chondrocytes.

Guided by the resulting mechanism, we engineered a dual‐action siMIF microsphere/quercetin hydrogel (SQH) designed to modulate senescence‐associated cell states while reducing local MIF expression through MIF‐siRNA delivery. Together, our data identify a senescence‐associated B cell–MIF axis that links joint aging to chondrocyte dysfunction and provide proof of concept that combined modulation of cellular senescence and MIF signaling can attenuate OA progression.

## 2 Results

### 2.1 Single‐Cell and Spatial Transcriptomic Analyses Reveal B‐Cell Functional Reprogramming and Proportional Imbalance During OA Progression and Joint Aging

To characterize cellular alterations associated with OA progression and joint aging, we performed single‐cell RNA sequencing on knee‐joint tissues collected from wild‐type control mice, naturally aged mice, and mice at multiple time points after OA induction. In parallel, spatial transcriptomic analysis was performed on representative tissue sections from control and OA‐24 W joints (Figure 1A). After quality control and integration, 246,877 cells were analyzed and annotated into eight major populations, including Granulocyte‐like cluster 1(GL1), B cells, chondrocytes, Granulocyte‐like cluster 2(GL2), T cells, erythrocytes, macrophages, and monocytes (Figure 1B). Granulocyte‐like cluster 1 was characterized by *Retnlg*, *Mmp8*, and *Mmp9* [28], whereas B cells expressed *Vpreb3*, *Cd79a*, and *Ebf1* [29]. Chondrocyte clusters expressed *S100a4*, *Fn1*, and *F13a1* [30], and Granulocyte‐like cluster 2 expressed Elane, Fcnb, and Prtn3 [31]. T cells were marked by *Ccl5*, *Ms4a4b*, and *Nkg7* [32], while erythrocytes by *Hbb‐bs*, *Hba‐a1*, and *Hba‐a2* [26]. Macrophage by the expression of *Vcam1*, *C1qa*, and *Hmox1* [33], and the monocyte cluster exhibited *Apoe*, *Gngt2*, and *Ear2* [34] (Figure 1C). Spatial transcriptomic compartment annotation further resolved major anatomical regions, including skeletal muscle/soft tissue, bone, synovium, meniscus, bone marrow, growth plate, cartilage, and subchondral bone (Figure 1D).

**FIGURE 1:**
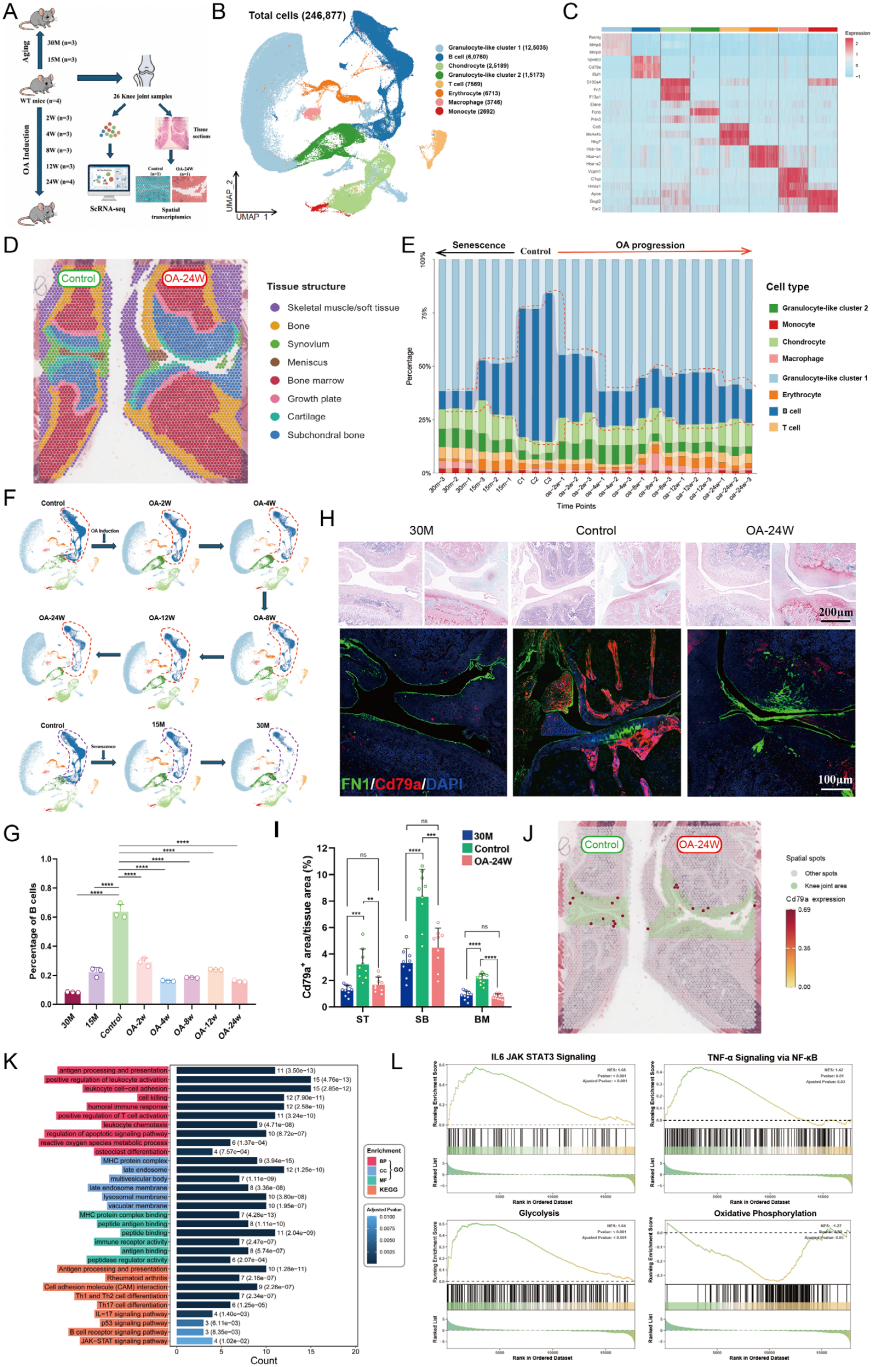
Single‐cell and spatial transcriptomic analyses reveal B‐cell functional reprogramming and proportional imbalance during OA progression and joint aging. (A) Schematic overview of the study design and analytical workflow. Twenty‐four knee‐joint samples (three mice per group) from wild‐type controls, naturally aged mice, and mice at 2, 4, 8, 12, or 24 weeks after OA induction were analyzed by scRNA‐seq. One additional control section and one OA‐24 W section were analyzed by spatial transcriptomics. (B) UMAP visualization of 246,877 cells annotated as Granulocyte‐like cluster 1, B cells, chondrocytes, Granulocyte‐like cluster 2, T cells, erythrocytes, macrophages, or monocytes. (C) Heatmap of representative marker‐gene expression across the annotated populations. (D) Spatial annotation of skeletal muscle/soft tissue, bone, synovium, meniscus, bone marrow, growth plate, cartilage, and subchondral bone. (E) Cell‐type proportions across individual scRNA‐seq samples. Each bar represents one individual mouse; red dashed outlines indicate the B‐cell fraction. (F) UMAP projections showing changes in major populations across OA progression and aging. Red dashed circles highlight B‐cell‐enriched regions; purple dashed circles highlight aging‐associated redistribution. (G) B‐cell proportions across Control, OA‐progression, and aged groups (n = 3 mice per group). Each point represents the B‐cell proportion from one individual mouse. (H) Representative H&E, Safranin O/Fast Green, and FN1/Cd79a immunofluorescence images from 30 M, control, and OA‐24 W joints. Scale bars: 200 µm (histology) and 100 µm (immunofluorescence). (I) Quantification of Cd79a‐positive area in the synovium (ST), subchondral bone (SB), and bone marrow (BM). Each point represents one individual mouse (n = 9 mice per group). Multiple microscopic fields within each anatomical compartment were averaged to obtain one mouse‐level value. ^*^ *p* < 0.05; ^**^ *p* < 0.01; ^***^ *p* < 0.001; ^****^ *p* < 0.0001; NS, not significant. (J) Spatial Cd79a expression in control and OA‐24 W sections. Colored spots indicate normalized expression; warmer colors indicate higher values. (K) GO and KEGG enrichment of B‐cell differentially expressed genes. Bar length indicates gene count, and color intensity indicates *p* value. (L) Representative GSEA plots for IL‐6–JAK–STAT3 signaling, TNF‐α signaling via NF‐κB, glycolysis, and oxidative phosphorylation.

We next examined changes in cellular composition across OA progression and joint aging. Stacked bar plots of individual scRNA‐seq samples demonstrated substantial remodeling of the major joint cell populations, with particularly prominent changes in the B‐cell population across aged and OA groups (Figure 1E). UMAP projections similarly showed progressive redistribution of major populations, including B‐cell‐enriched regions, during OA progression and aging (Figure 1F). B‐cell abundance was lower in aged and late‐stage OA joints than in healthy controls (Figure 1G).

To further validate the single‐cell findings at the tissue level, we performed histological and immunofluorescence analyses of knee joints from representative 30‐month‐old (30 M), control, and OA‐24 W mice. As Cd79a is a well‐established B‐cell marker, we used Cd79a staining and expression to assess B‐cell abundance in joint tissues [35]. H&E and Safranin O/Fast Green staining confirmed cartilage damage and matrix loss in aged and OA joints. Immunofluorescence showed reduced Cd79a‐positive area together with increased FN1 deposition in diseased tissues (Figure 1H). Compartment‐specific quantification showed lower Cd79a‐positive area in 30 M and OA‐24 W joints than in controls within the synovium, subchondral bone, and bone marrow (Figure 1I), and spatial transcriptomics showed a corresponding reduction in Cd79a signal in the OA‐24 W section (Figure 1J).

Having validated the reduction in B‐cell abundance at the tissue level, we next examined disease‐associated transcriptional programs of B‐cell population using GO and KEGG enrichment analyses. B‐cell differentially expressed genes were enriched for antigen processing and presentation, leukocyte activation, regulation of T‐cell activation, and inflammatory pathways, including IL‐17 and JAK–STAT signaling, consistent with an inflammatory activation state in OA and aged joints. Enrichment of p53 signaling, regulation of apoptotic signaling, reactive oxygen species metabolism, and lysosome‐related processes additionally suggested cellular stress and senescence‐associated remodeling (Figure 1K). In line with these findings, gene set enrichment analysis demonstrated positive enrichment of IL‐6–JAK–STAT3 signaling, TNF‐α signaling via NF‐κB, and glycolysis, together with negative enrichment of oxidative phosphorylation, indicating coordinated inflammatory and metabolic reprogramming of B cells during OA progression and joint aging (Figure 1L). These data establish a single‐cell and spatial transcriptomics atlas of OA and aged mouse joints and identify B‐cell functional reprogramming and proportional imbalance as prominent features of disease progression and joint aging.

### 2.2 Senescence‐Associated B‐Cell Transcriptional States Accompany Chondrocyte Degeneration During OA Progression and Joint Aging

To define B‐cell remodeling during OA progression and joint aging, we reclustered B cells from the integrated single‐cell atlas. t‐SNE visualization resolved 15 transcriptionally distinct clusters, which were annotated into 12 B‐cell subtypes or states, including immature B cells [36], naive B cells [37], memory B cells [38], germinal‐center dark‐zone B cells [39, 40, 41], pro‐B cells [41, 42], marginal‐zone B cells [43], plasma cells [44], plasmablasts [45], cycling pre‐B cells [36], interferon‐induced B cells [46], Top2a‐expressing B cells [41], and unclassified B cells (Figure 2A). These annotations were supported by representative subtype‐specific marker genes, as shown in the heatmap (Figure 2B), with the cluster‐level annotation summary and supporting markers provided in Table S2. To assess inter‐mouse consistency in B‐cell subtype representation, we further examined the distribution and relative composition of these subtypes across individual animals (Figures S1 and S2).

**FIGURE 2:**
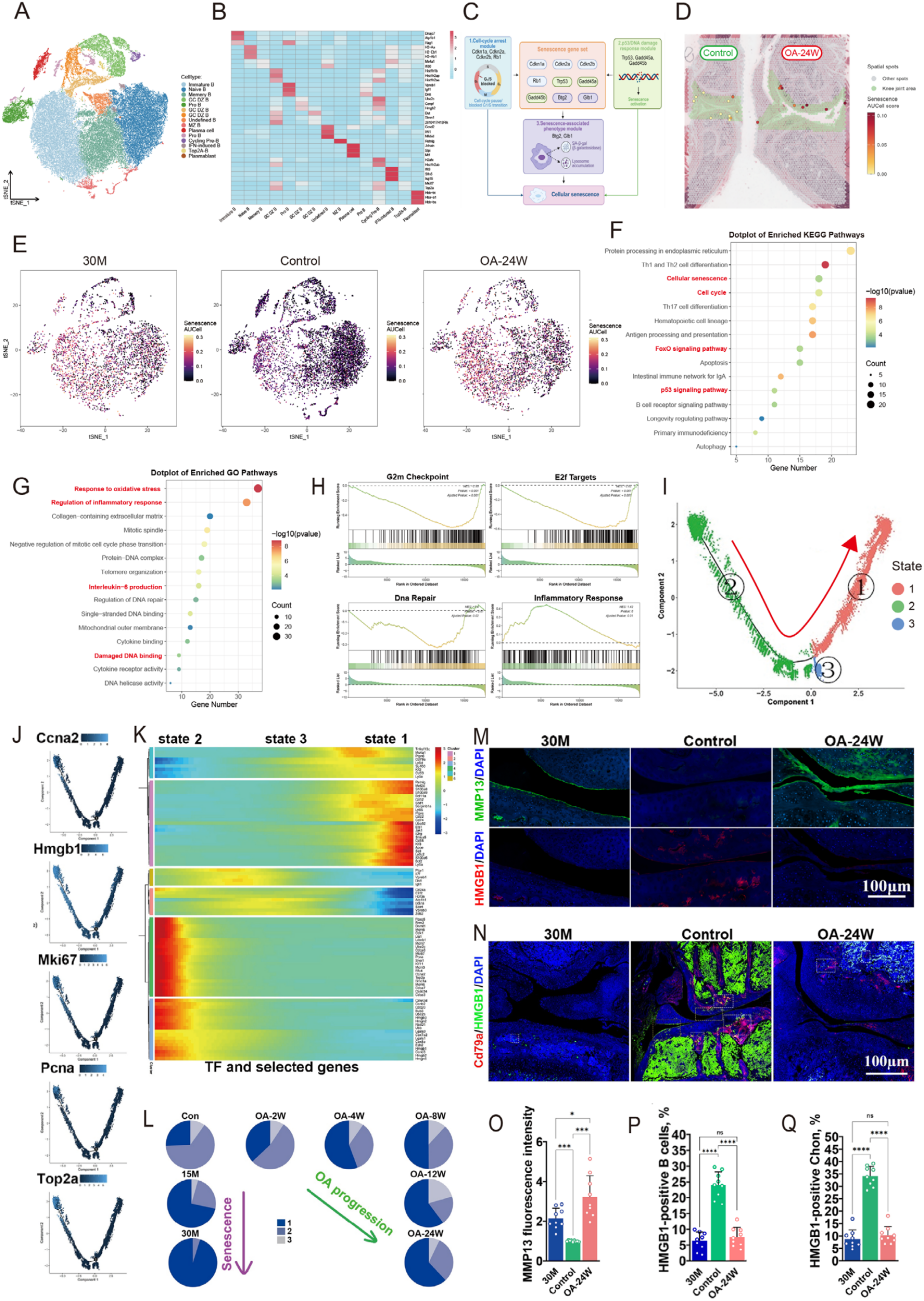
B cells shift toward senescence‐associated states with concomitant chondrocyte degeneration in OA and aged joints. (A) t‐SNE visualization of B‐cell subtypes identified in mouse joint tissues. (B) Heatmap of representative marker‐gene expression across the annotated B‐cell subtypes. (C) Complete nine‐gene senescence‐associated transcriptional gene set used for AUCell scoring. Genes are grouped according to their biological associations with cell‐cycle arrest, p53/DNA‐damage responses, and senescence‐associated cellular phenotypes. (D) Spatial visualization of senescence AUCell scores in control and OA‐24 W knee sections. Colored spots indicate scores; warmer colors indicate higher values. (E) AUCell scores projected onto t‐SNE embeddings of B cells from 30 M, control, and OA‐24 W groups. (F) KEGG enrichment analysis of B‐cell differentially expressed genes. (G) GO enrichment analysis of B‐cell differentially expressed genes. (H) GSEA plots showing representative pathways in B cell. (I) Pseudotime trajectory showing three inferred B‐cell states and the selected direction from State 2 through State 3 toward State 1. (J) Pseudotime expression dynamics of representative cell‐cycle‐ and proliferation‐associated genes, including Ccna2, Mki67, Pcna, Top2a, and Hmgb1. (K) Heatmap of transcription factors and selected genes dynamically regulated along the B‐cell trajectory. (L) Distribution of B‐cell states across control, OA‐progression, and aged groups. (M) Representative MMP13 and HMGB1 immunofluorescence in 30 M, control, and OA‐24 W joints. Scale bar: 100 µm. (N) Representative Cd79a/HMGB1 immunofluorescence in 30 M, control, and OA‐24 W joints. Dashed rectangles indicate regions containing CD79A‐positive B cells. Scale bar: 100 µm. (O–Q) Quantification of MMP13 fluorescence intensity (O), HMGB1‐positive B cells (P), and HMGB1‐positive chondrocytes (Q) (n = 9 mice per group). Each point represents one individual mouse. ^*^ *p* < 0.05; ^**^ *p* < 0.01; ^***^ *p* < 0.001; ^****^ *p* < 0.0001; NS, not significant.

Building on the stress‐ and senescence‐associated signatures identified in Figure 1, we defined a nine‐gene senescence‐associated transcriptional gene set comprising genes related to cell‐cycle arrest (Cdkn1a, Cdkn2a, Cdkn2b, and Rb1), p53/DNA‐damage responses (Trp53, Gadd45a, and Gadd45b), and senescence‐associated cellular phenotypes (Btg2 and Glb1) (Figure 2C). These nine genes constituted the complete gene set used for AUCell scoring; their functional categories, rationale for inclusion, and supporting references are provided in Table S1. AUCell was used to quantify enrichment of this predefined gene set in individual B cells, with higher AUCell scores indicating greater enrichment of the senescence‐associated transcriptional program.

Spatial mapping of the previously defined Cd79a‐positive, B‐cell‐associated spots in the representative sections showed that the OA‐24 W section contained a broader distribution of spots with high senescence‐associated AUCell scores than the Control section (Figure 2D). Because spatial transcriptomics was performed on one section per condition, this spatial pattern was interpreted descriptively as representative anatomical context rather than as evidence of a biologically replicated group‐level difference. In parallel, B cells from the 30 M and OA‐24 W scRNA‐seq datasets exhibited higher senescence‐associated transcriptional activity than those from the healthy Control dataset (Figure 2E). Together, these observations provide supportive evidence for senescence‐associated transcriptional remodeling of B cells during OA progression and joint aging.

Consistent with the AUCell‐based senescence scoring in representative groups, enrichment analyses of B‐cell DEGs from all OA and aged groups further supported senescence‐associated inflammatory changes. KEGG analysis showed enrichment of cellular senescence, cell cycle, FoxO signaling, and p53 signaling pathways (Figure 2F), while GO analysis highlighted response to oxidative stress, regulation of inflammatory response, interleukin‐6 production, and damaged DNA binding (Figure 2G). GSEA further showed that G2M checkpoint, E2F targets, and DNA repair pathways were significantly downregulated, whereas the inflammatory response pathway was significantly upregulated (Figure 2H). Collectively, these enrichment results indicate that B cells in OA and aged joints exhibit senescence‐associated inflammatory transcriptional features characterized by suppressed proliferative and genome‐maintenance programs together with enhanced inflammatory, oxidative‐stress, and DNA‐damage‐response features.

To further resolve the dynamic remodeling of B cells during OA progression and joint aging, we ordered B cells along a pseudotime trajectory comprising three inferred states (Figure 2I). With State 2 selected as the root, the trajectory extended through State 3 toward State 1. Along this ordered trajectory, cell‐cycle and proliferation‐associated genes, including *Ccna2*, *Mki67*, *Pcna*, and *Top2a*, showed reduced expression toward State 1, indicating a decline in proliferative activity (Figure 2J,K). In parallel, *Hmgb1* also decreased along the State 1‐associated trajectory, consistent with loss of HMGB1‐positive cells in aged and OA joints. Together with the preceding enrichment results, these gene‐expression dynamics support State 1 as a senescence‐associated B‐cell state. Notably, State 1 B cells were more prominently represented in aged and OA joints than in healthy controls, with differences in their relative representation across the OA time points (Figure 2L). These results suggest that OA progression and joint aging are associated with increased representation of a low‐proliferative and senescence‐associated B‐cell transcriptional state. Importantly, this pseudotime ordering represents an inferred transcriptional relationship among B‐cell states rather than direct evidence of a directional in vivo lineage transition.

To validate these findings, we performed immunofluorescence staining of knee joint sections from control, 30 M, and OA‐24 W mice. Compared with controls, 30 M and OA‐24 W joints showed increased MMP13 staining and significantly higher MMP13 fluorescence intensity (Figure 2M,O). In contrast, HMGB1‐positive B cells and HMGB1‐positive chondrocytes were reduced in 30 M and OA‐24 W joints (Figure 2N,P,Q). The reduction in HMGB1‐positive B cells was consistent with the pseudotime‐associated decrease in Hmgb1 expression, whereas increased MMP13 indicated enhanced cartilage‐degenerative activity. Together, these findings indicate that OA progression and joint aging are characterized by a shift toward senescence‐associated B‐cell states accompanied by cartilage degeneration.

### 2.3 MIF Signaling is Enhanced Between Senescence‐Associated B Cells and Chondrocytes in OA and Aged Joints

Given that senescence‐associated B‐cell remodeling was accompanied by cartilage degeneration, we next investigated whether specific intercellular signaling pathways connected these processes. CellChat analysis of representative Control, 30 M, and OA‐24 W groups identified MIF as one of the pathways showing increased predicted intercellular communication in the aged and late‐stage OA datasets. Predicted MIF‐related interactions increased among several populations, with notable predicted B cell–chondrocyte communication in 30 M and OA‐24 W joints (Figure 3A).

**FIGURE 3:**
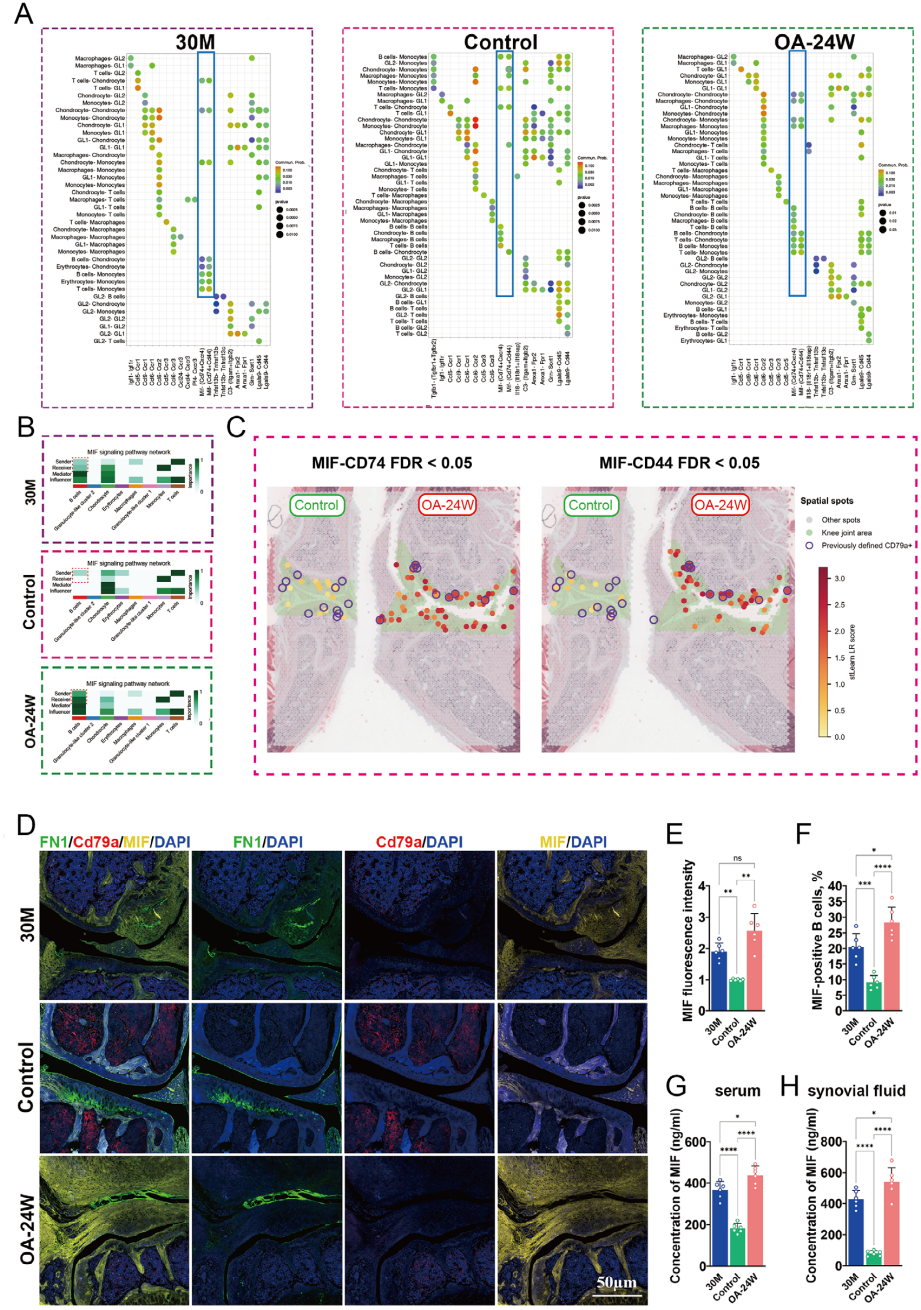
Enhanced MIF signaling is associated with senescence‐associated B‐cell remodeling and predicted B cell–chondrocyte communication in OA and aged joints. (A) Predicted ligand–receptor communication among major joint‐cell populations in 30 M, control, and OA‐24 W groups. Blue rectangles highlight MIF‐related interactions; dot color indicates communication probability. (B) CellChat network‐centrality analysis of MIF signaling showing sender, receiver, mediator, and influencer activity. (C) Spatially predicted MIF‐associated interaction scores for the CD74/CD44 receptor complex in Control and OA‐24 W sections. Purple outlines indicate *Cd79a*‐positive B‐cell‐associated spots; colored spots indicate mean interaction scores meeting FDR < 0.05. (D) Representative FN1, Cd79a, and MIF immunofluorescence in 30 M, control, and OA‐24 W joints. Scale bar: 50 µm. (E–H) Quantification of MIF fluorescence intensity (E), proportion of MIF‐positive B cells (F), serum MIF concentration (G), and synovial‐fluid MIF concentration (H) (n = 6 mice per group). Each point represents one individual mouse. ^*^ *p* < 0.05; ^**^ *p* < 0.01; ^***^ *p* < 0.001; ^****^ *p* < 0.0001; NS, not significant.

Network‐centrality analysis further showed higher predicted sender and receiver centrality scores for B cells within the MIF communication network in the 30 M and OA‐24 W datasets (Figure 3B), suggesting potential involvement of B cells in MIF‐associated intercellular communication.

To examine the spatial distribution of the computationally predicted MIF‐associated interactions, we mapped MIF–CD74 and MIF–CD44 interaction scores in the representative control and OA‐24 W sections. In the sections analyzed, the OA‐24 W section displayed more spots with high predicted interaction scores and greater apparent spatial overlap or adjacency between MIF‐associated interaction hotspots and B‐cell‐associated regions with higher senescence‐associated scores (Figure 3C). These observations therefore provide anatomical context for the predicted involvement of MIF‐associated CD74/CD44 receptor‐complex signaling in regions associated with B‐cell remodeling and cartilage degeneration.

Immunofluorescence confirmed increased MIF staining in 30 M and OA‐24 W joints, accompanied by increased FN1 deposition and altered B‐cell distribution (Figure 3D,E). The proportion of MIF‐positive B cells was also higher in aged and OA joints (Figure 3F).

To further validate MIF upregulation at the systemic and intra‐articular levels, we measured MIF concentrations in serum and synovial fluid. MIF concentrations were elevated in both the serum and synovial fluid of 30 M and OA‐24 W mice compared with Control mice, indicating systemic and local upregulation of MIF during joint aging and OA progression (Figure 3G,H).

Collectively, these results support increased MIF abundance together with enhanced computationally predicted MIF‐associated communication in OA and aged joints. In particular, the accumulation of MIF‐positive B cells, enhanced predicted MIF‐related B cell–chondrocyte communication, and the spatial association of predicted MIF–CD74 and MIF–CD44 interaction activity with senescence‐associated B‐cell and cartilage regions support a potential MIF‐centered connection between senescence‐associated B‐cell remodeling and chondrocyte degeneration. These findings identify the senescence‐associated B cell–MIF axis as a candidate mediator of B cell–chondrocyte crosstalk.

### 2.4 Genetic MIF Deficiency Attenuates OA Progression and Senescence‐Associated B‐Cell Alterations In Vivo

Given the enhanced MIF signaling observed in OA and aged joints, we next examined whether genetic MIF deficiency attenuates OA progression in vivo. DMM‐induced OA was established in wild‐type (WT) and *Mif^−/−^* mice, and knee joints were analyzed at 8 and 24 weeks after surgery. Baseline phenotyping showed that *Mif^−/−^* mice had lower body weight and shorter body length than WT mice (Figure 4A–C). Serum MIF concentrations were comparable between WT and *Mif ^+/−^* mice, whereas *Mif ^−/−^* mice showed nearly undetectable serum MIF levels, confirming effective MIF deletion (Figure 4D).

**FIGURE 4:**
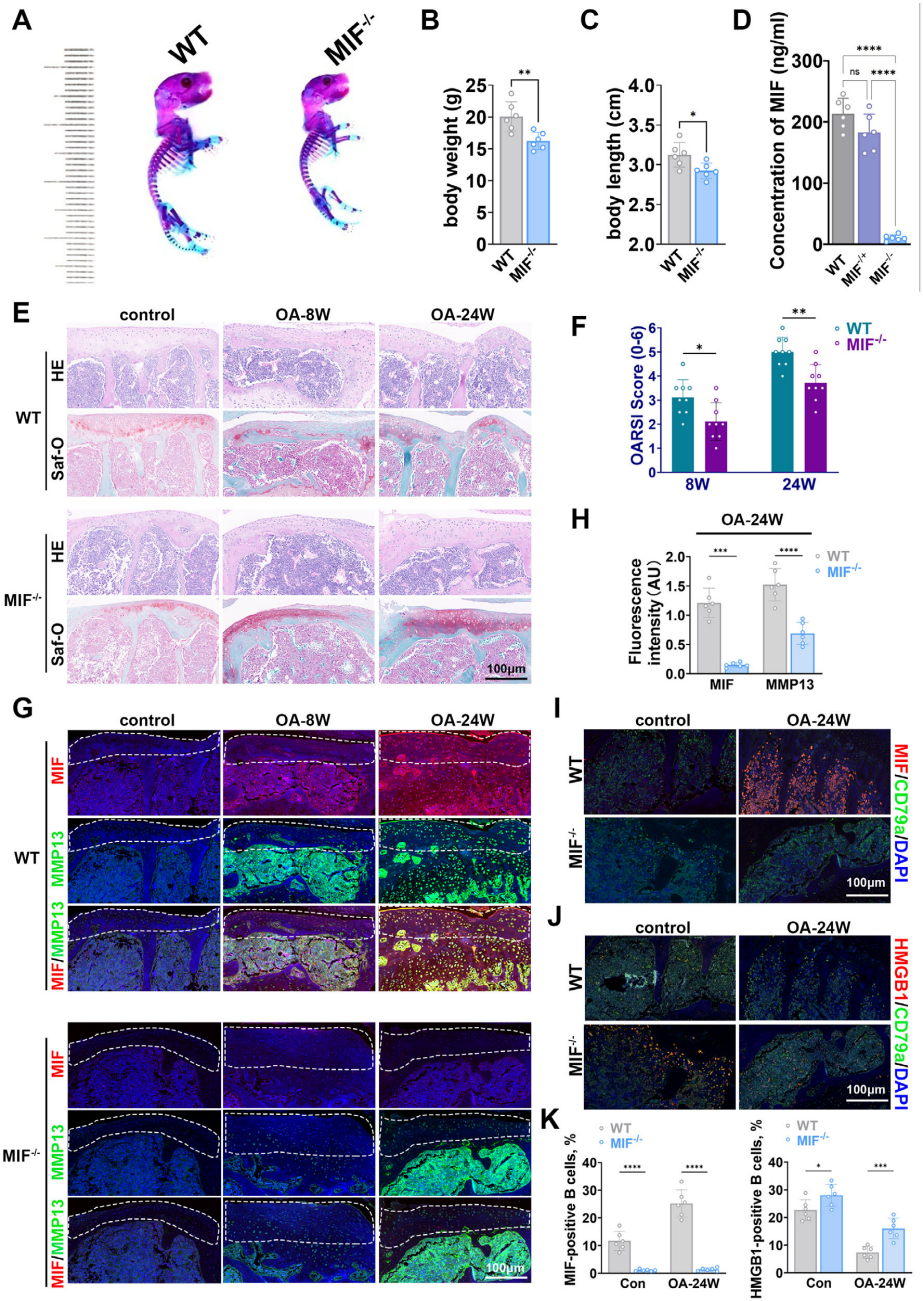
MIF deficiency protects against OA progression and B‐cell senescence‐associated cartilage degeneration in vivo. (A) Skeletal staining of WT and *Mif^−/−^* mice. Alizarin Red and Alcian Blue indicate bone and cartilage, respectively. (B, C) Body weight and body length in WT and Mif^−/−^ mice (n = 6 per group). ^*^ *p* < 0.05; ^**^ *p* < 0.01; ^***^ *p* < 0.001; ^****^ *p* < 0.0001; NS, not significant. (D) Serum MIF concentrations in WT, *Mif^+/−^*, and *Mif^−/−^* mice (n = 6 per group). ^*^ *p* < 0.05; ^**^ *p* < 0.01; ^***^ *p* < 0.001; ^****^ *p* < 0.0001; NS, not significant. (E) H&E and Safranin O/Fast Green staining of WT and *Mif^−/−^* knees under control, OA‐8 W, and OA‐24 W conditions. Scale bar: 100 µm. (F) Terminal OARSI histological scores at 8 and 24 weeks after DMM (n = 9 mice per genotype at each terminal time point). Each point represents one individual mouse. The 8‐ and 24‐week groups comprised independent terminal cohorts and were not treated as repeated measurements. ^*^ *p* < 0.05; ^**^ *p* < 0.01; ^***^ *p* < 0.001; ^****^ *p* < 0.0001; NS, not significant. (G) MIF and MMP13 immunofluorescence in WT and *Mif^−/−^* knees. Dashed lines indicate the cartilage surface. (H) MIF and MMP13 fluorescence intensity in OA‐24 W joints (n = 6 per group). AU, arbitrary units. ^*^ *p* < 0.05; ^**^ *p* < 0.01; ^***^ *p* < 0.001; ^****^ *p* < 0.0001; NS, not significant. (I, J) Cd79a/MIF and Cd79a/HMGB1 immunofluorescence in WT and *Mif^−/−^* knees under control and OA‐24 W conditions. Scale bar: 100 µm. (K) Proportions of MIF‐positive and HMGB1‐positive B cells (n = 6 mice per group). Each point represents one individual mouse. ^*^ *p* < 0.05; ^**^ *p* < 0.01; ^***^ *p* < 0.001; ^****^ *p* < 0.0001; NS, not significant.

Histological analysis of H&E and Safranin O‐stained knees showed intact cartilage in both WT and *Mif^−/−^* control mice. At 8 weeks after OA induction, WT mice exhibited early cartilage damage, whereas *Mif^−/−^* mice showed milder cartilage degeneration. By 24 weeks, WT mice developed extensive cartilage erosion and matrix loss, while *Mif^−/−^* mice preserved better cartilage integrity (Figure 4E). Consistently, terminal OARSI histological scores were significantly lower in *Mif^−/−^* mice than in WT mice at both 8 and 24 weeks after OA induction, indicating that loss of MIF reduced cartilage damage and attenuated OA progression (Figure 4F).

Immunofluorescence staining further showed increased MIF and MMP13 signals in OA joints from WT mice, particularly at 24 weeks, whereas *Mif^−/−^* mice exhibited nearly absent MIF staining and reduced MMP13 expression (Figure 4G,H). These results suggest that loss of MIF reduces MMP13‐associated cartilage degradation during OA progression. We further examined MIF and HMGB1 expression in B cells within joint tissues. WT OA joints showed an increased proportion of MIF‐positive B cells and a reduced proportion of HMGB1‐positive B cells. In contrast, these changes were partially reversed in *Mif^−/−^* OA joints, with fewer MIF‐positive B cells and restored HMGB1‐positive B cells compared with WT OA joints (Figure 4I–K). Together, these in vivo findings show that constitutive global MIF deficiency is associated with reduced DMM‐induced cartilage damage, lower MMP13 expression, and altered senescence‐associated B‐cell markers. Because MIF was deleted systemically throughout life, these findings do not attribute the protective phenotype specifically to B‐cell‐derived MIF and do not exclude developmental, systemic immune, or biomechanical contributions. These findings support the in vivo relevance of MIF in OA and prompted us to further examine whether B‐cell‐associated MIF contributes to chondrocyte degeneration.

### 2.5 Senescence‐Associated B Cells Promote Chondrocyte Senescence and Degenerative Remodeling Through MIF‐Dependent Signaling In Vitro

To determine whether the in vivo protection conferred by MIF deficiency involved a B cell–chondrocyte mechanism, we investigated whether OA‐ and aging‐associated B cells promote chondrocyte degeneration through MIF‐dependent signaling. We established a Transwell co‐culture system using primary chondrocytes and mouse joint‐derived B cells isolated by magnetic bead sorting (Figure 5A). B cells were obtained from Control, OA‐8 W, OA‐24 W, *Mif^−/−^*, and 15‐month‐old (15 M) mice. Before co‐culture, we first characterized the senescence phenotype and MIF production of these B‐cell populations. Compared with Control‐derived B cells, OA‐8 W, OA‐24 W, and 15M‐derived B cells exhibited increased P16 and P21 expression, with the most pronounced upregulation observed in the OA‐24 W and 15 M groups (Figure 5B–D). SA‐β‐gal staining further confirmed a more pronounced senescent phenotype in OA‐24W‐ and 15M‐derived B cells, whereas SA‐β‐gal positivity in *Mif^−/−^* B cells was comparable to that in Control‐derived B cells (Figure 5B,E). MIF concentrations in B‐cell culture supernatants were markedly elevated in the OA‐8 W, OA‐24 W, and 15 M groups, whereas *Mif^−/−^* B cells showed minimal MIF secretion (Figure 5F). Together, these results indicate that OA progression and aging are associated with the accumulation of senescence‐associated B cells with increased MIF secretion.

**FIGURE 5:**
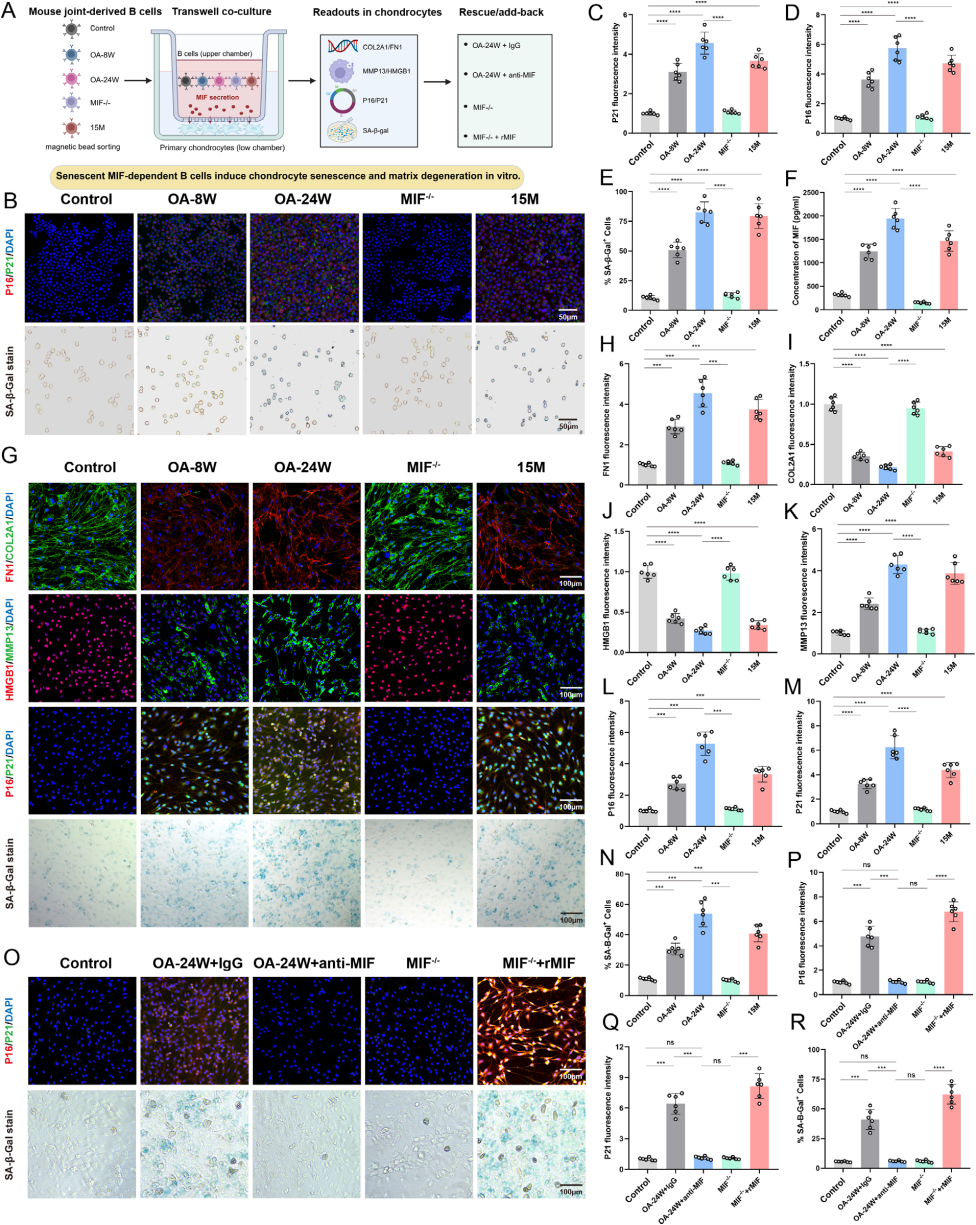
Senescence‐associated B cells promote MIF‐dependent chondrocyte senescence and degenerative remodeling in vitro. (A) Experimental design. Joint‐derived B cells from Control, OA‐8 W, OA‐24 W, *Mif^−/−^*, and 15 M mice were magnetically isolated and co‐cultured with primary chondrocytes in Transwell inserts. MIF neutralization and recombinant‐MIF add‐back were also tested. (B) P16/P21 immunofluorescence and SA‐β‐gal staining of joint‐derived B cells. (C–F) Quantification of B‐cell P21 (C), P16 (D), SA‐β‐gal positivity (E), and MIF secretion (F) (n = 6 mice per group). ^*^ *p* < 0.05; ^**^ *p* < 0.01; ^***^ *p* < 0.001; ^****^ *p* < 0.0001; NS, not significant. (G) COL2A1/FN1, MMP13/HMGB1, and P16/P21 immunofluorescence and SA‐β‐gal staining of chondrocytes after B‐cell co‐culture. (H–N) Quantification of chondrocyte FN1 (H), COL2A1 (I), HMGB1 (J), MMP13 (K), P16 (L), P21 (M), and SA‐β‐gal positivity (N) (n = 6 mice per group). ^*^ *p* < 0.05; ^**^ *p* < 0.01; ^***^ *p* < 0.001; ^****^ *p* < 0.0001; NS, not significant. (O) MIF neutralization and add‐back experiments. Chondrocytes were co‐cultured with Control B cells, OA‐24 W B cells plus IgG, OA‐24 W B cells plus anti‐MIF antibody, *Mif^−/−^* B cells, or *Mif^−/−^* B cells plus recombinant MIF. (P–R) Quantification of P16 (P), P21 (Q), and SA‐β‐gal‐positive chondrocytes (R) (n = 6 mice per group). ^*^ *p* < 0.05; ^**^ *p* < 0.01; ^***^ *p* < 0.001; ^****^ *p* < 0.0001; NS, not significant.

After Transwell co‐culture, chondrocytes exposed to OA‐24W‐ or 15M‐derived B cells showed increased FN1 and MMP13 expression, together with reduced COL2A1 and HMGB1 expression (Figure 5G–K), indicating loss of chondrocyte homeostasis and enhanced fibrotic and degenerative remodeling. In contrast, chondrocytes co‐cultured with *Mif^−/−^* B cells largely retained COL2A1 and HMGB1 expression and showed reduced FN1 and MMP13 induction, suggesting that MIF deficiency attenuated the pro‐degenerative effect of OA and aging‐associated B cells. Consistently, OA‐24 W and 15M‐derived B cells induced pronounced chondrocyte senescence, as shown by increased P16 and P21 expression and elevated SA‐β‐gal positivity, whereas *Mif^−/−^* B cells induced substantially milder senescence‐related changes (Figure 5G,L–N).

To test whether MIF is functionally required for OA‐derived B‐cell‐induced chondrocyte senescence, we performed MIF blockade and add‐back experiments. Compared with chondrocytes co‐cultured with OA‐24W‐derived B cells in the presence of IgG control, treatment with an anti‐MIF neutralizing antibody significantly reduced P16 and P21 expression and decreased the proportion of SA‐β‐gal‐positive chondrocytes (Figure 5O–R). Anti‐MIF treatment reduced these senescence markers to levels comparable to those observed in Control or *Mif^−/−^* B‐cell co‐cultures. Conversely, recombinant MIF add‐back restored the ability of *Mif^−/−^* B cells to promote chondrocyte senescence, resulting in increased P16 and P21 expression and elevated SA‐β‐gal positivity (Figure 5O–R). Together, these loss‐of‐function, neutralization, and add‐back experiments support a role for extracellular MIF in mediating the chondrocyte senescence‐related and degenerative changes induced by OA‐ and aging‐associated joint‐derived B cells in vitro.

### 2.6 Construction and Characterization of a Dual‐Action SQH Hydrogel Guided by the Senescence‐Associated B Cell–MIF Axis

Together, our single‐cell, spatial, in vivo, and in vitro analyses support a pathological model in which OA progression and joint aging drive senescence‐associated B‐cell remodeling. In the diseased joint, cartilage matrix destruction and antigenic stimulation are associated with B‐cell functional changes, including reduced proliferative features and enhanced inflammatory activation. These findings suggest that senescence‐associated B‐cell remodeling is accompanied by increased MIF signaling and may contribute to chondrocyte senescence‐related and degenerative changes (Figure 6A). These findings provided the rationale for developing a composite therapeutic strategy aimed at modulating senescence‐associated cellular changes while reducing local MIF expression.

**FIGURE 6:**
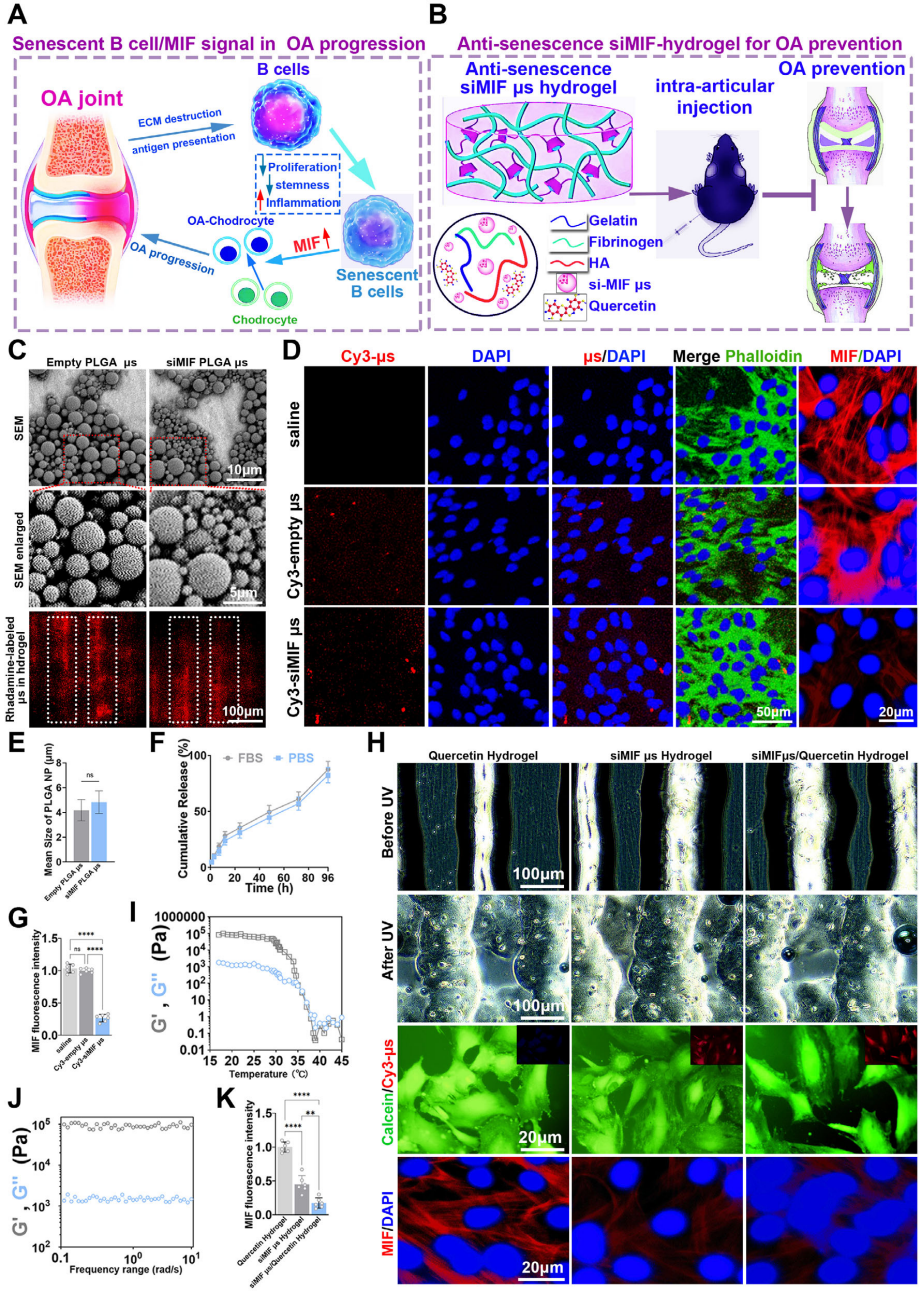
Construction and characterization of a dual‐action siMIF microsphere/quercetin hydrogel. (A) Schematic illustration of the senescent B cell–MIF–chondrocyte axis during OA progression. (B) Design of the siMIF microsphere/quercetin hydrogel (SQH). (C) SEM images of empty and siMIF‐loaded PLGA microspheres and microscopy of rhodamine‐labeled microspheres distributed within the hydrogel. (D) Fluorescence micrographs showing siMIF microsphere‐derived signals in close spatial association with phalloidin‐stained chondrocytes, together with MIF immunofluorescence staining in treated chondrocytes. (E) Mean diameters of empty and siMIF‐loaded microspheres from 12 independently prepared microsphere batches per formulation (n = 12 independent batches per group). NS, not significant. (F) Cumulative in vitro release profile of Cy3‐labeled MIF‐siRNA from PLGA microspheres in PBS and FBS at 37°C over 96 h (n = 12 independently prepared microsphere batches). Data are presented as mean ± SD. (G) Quantification of MIF immunofluorescence intensity in (D) (n = 6 chondrocyte donor mice per group). ^*^ *p* < 0.05; ^**^ *p* < 0.01; ^***^ *p* < 0.001; ^****^ *p* < 0.0001; NS, not significant. (H) Representative images of different hydrogel formulations, including quercetin hydrogel (QH), siMIF microsphere hydrogel (SH), and siMIF microsphere/quercetin hydrogel (SQH). Hydrogel shape fidelity after UV crosslinking, chondrocyte morphology, siMIF microsphere‐derived fluorescence associated with chondrocytes, and MIF immunofluorescence staining are shown. (I) Temperature‐dependent rheological analysis of the pre‐crosslinked SQH formulation. Storage modulus (G′) and loss modulus (G″) were measured from 15°C to 45°C. Rheological profiles were obtained from 12 independently prepared SQH samples (n = 12) and were treated as descriptive material‐characterization data. (J) Frequency‐sweep rheological analysis of the final UV‐crosslinked SQH at 17°C. Storage and loss moduli were recorded over an angular‐frequency range of 0.1–10 rad/s using 12 independently prepared SQH samples (n = 12). (K) Quantification of MIF immunofluorescence intensity in (H) (n = 6 chondrocyte donor mice per group). ^*^ *p* < 0.05; ^**^ *p* < 0.01; ^***^ *p* < 0.001; ^****^ *p* < 0.0001; NS, not significant.

Guided by this senescence‐associated B cell–MIF–chondrocyte axis, we selected quercetin to modulate senescence‐associated phenotypes during OA progression and incorporated MIF‐siRNA‐loaded PLGA microspheres to reduce MIF expression within the osteoarthritic joint. We therefore constructed a composite siMIF microsphere/quercetin hydrogel (SQH) by incorporating both components into the hydrogel matrix (Figure 6B). This dual‐action system was designed to combine quercetin‐mediated modulation of senescence‐associated phenotypes with local MIF reduction through MIF‐siRNA delivery.

Scanning electron microscopy (SEM) images showed that both empty and siMIF‐loaded PLGA microspheres exhibited a uniform spherical morphology, with particle diameters mainly ranging from 3 to 6 µm and mean diameters below 5 µm (Figure 6C–E). In vitro release analysis showed gradual release of siMIF from PLGA microspheres over the measured 96‐h interval (Figure 6F). Rhodamine‐labeled siMIF microspheres were evenly distributed within the crosslinked hydrogel, confirming successful microsphere incorporation into the hydrogel matrix (Figure 6C). Fluorescence imaging showed siMIF microsphere‐derived signals in close spatial association with phalloidin‐stained chondrocytes, while siMIF microsphere treatment reduced MIF immunofluorescence in cultured chondrocytes (Figure 6D,G).

Before UV crosslinking, the SQH formulation exhibited thermoresponsive behavior, appearing more solid‐like below 25°C and becoming liquid‐like at 37°C and above. Temperature‐sweep rheological analysis of the pre‐crosslinked SQH showed a temperature‐dependent decrease in storage modulus (G′), with a crossover between G′ and loss modulus (G″) at approximately 35°C, consistent with a gel‐to‐solution transition during heating (Figure 6I). The formulation was subsequently crosslinked under UV light to generate the final SQH used for intra‐articular injection, which maintained good structural integrity under light microscopy (Figure 6H). Chondrocytes co‐cultured with different hydrogel formulations showed no obvious morphological abnormalities, and fluorescence imaging showed siMIF microsphere‐derived signals in close spatial association with cultured chondrocytes (Figure 6H). Immunofluorescence staining further showed reduced MIF signal in the SQH co‐culture group (Figure 6H,K). Together, these results demonstrate the thermoresponsive properties of SQH, its compatibility with cultured chondrocytes under the examined conditions, local siMIF delivery, and reduced MIF immunofluorescence following SQH treatment.

### 2.7 Dual‐Action Modulation of the Senescence‐Associated B Cell–MIF Axis by SQH Hydrogel Attenuates OA Progression

Based on the dual‐action SQH hydrogel constructed and characterized above, we next evaluated its capacity to attenuate MIF‐associated chondrocyte degeneration and OA progression in vitro and in vivo.

In vitro, OA chondrocytes were treated with different hydrogel formulations, including quercetin hydrogel (QH), siMIF microsphere hydrogel (SH), and the combined siMIF microsphere/quercetin hydrogel (SQH). Among the tested formulations, SQH exhibited the most favorable overall profile across the evaluated OA‐associated chondrocyte markers. SQH markedly reduced MIF and MMP13 expression compared with untreated OA chondrocytes and QH‐treated cells (Figure 7A–D). SQH treatment also decreased P16 expression and restored HMGB1 expression in OA chondrocytes (Figure 7B,E,F), indicating that the combined quercetin/siMIF formulation attenuated MIF‐associated catabolic and senescence‐related changes in vitro.

**FIGURE 7:**
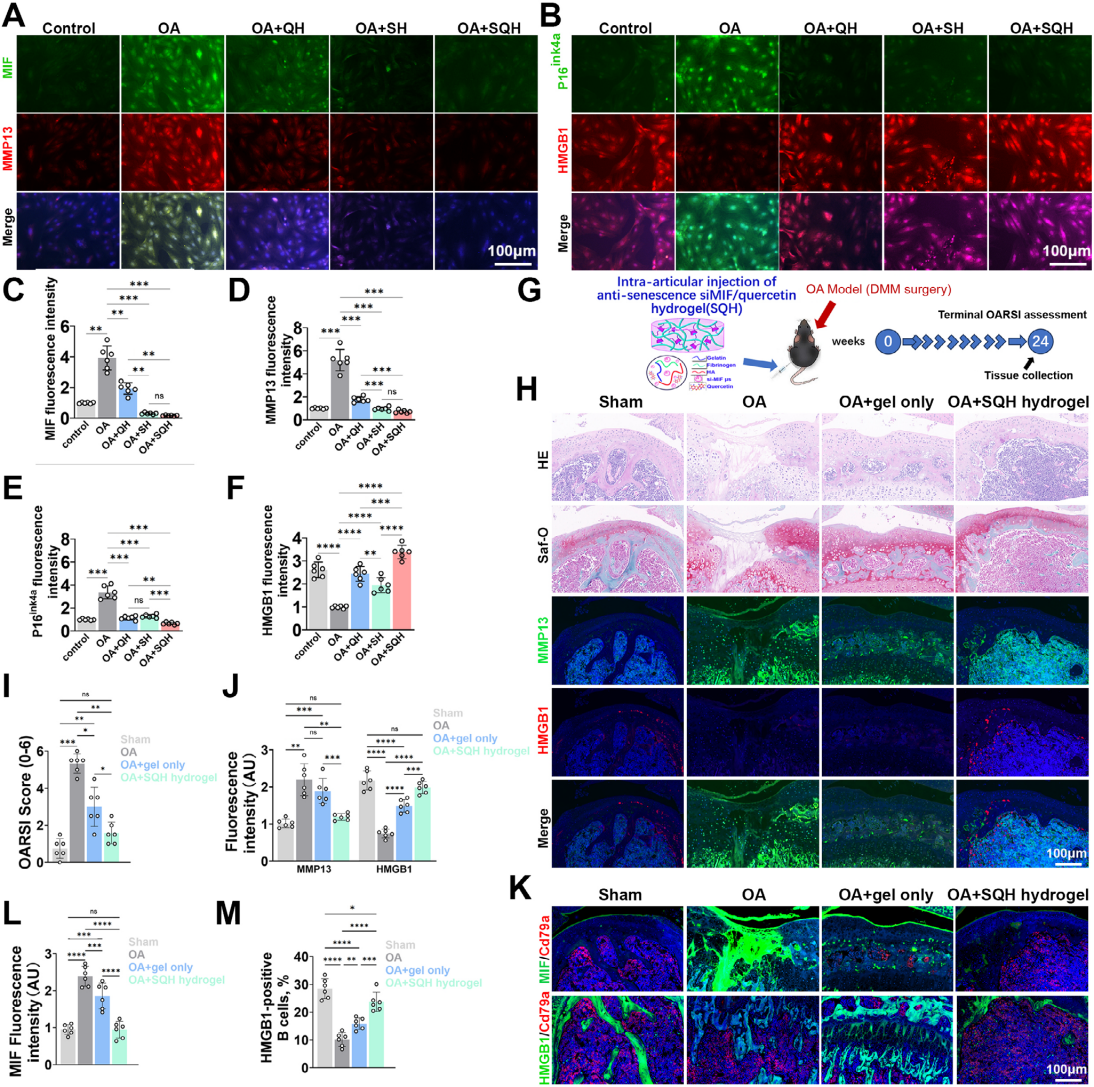
SQH hydrogel attenuates OA‐associated cartilage degeneration and modulates senescence‐associated B cell–MIF‐related changes. (A) MIF and MMP13 immunofluorescence in control and OA chondrocytes treated with quercetin hydrogel (QH), siMIF microsphere hydrogel (SH), or siMIF microsphere/quercetin hydrogel (SQH). (B) Immunofluorescence staining of P16^INK4a^ and HMGB1 in chondrocytes from different treatment groups. Control or OA chondrocytes were treated with different hydrogel formulations. (C–F) Quantification of MIF, MMP13, P16, and HMGB1 fluorescence (n = 6 biological replicates per group). ^*^ *p* < 0.05; ^**^ *p* < 0.01; ^***^ *p* < 0.001; ^****^ *p* < 0.0001; NS, not significant. (G) Experimental timeline for intra‐articular hydrogel or SQH treatment after DMM and analysis at 24 weeks. (H) H&E and Safranin O/Fast Green staining and MMP13/HMGB1 immunofluorescence in Sham, OA, OA + hydrogel, and OA + SQH joints. Scale bar: 100 µm. (I) Terminal OARSI histological scores (n = 6 mice per group). Each point represents one individual mouse. ^*^ *p* < 0.05; ^**^ *p* < 0.01; ^***^ *p* < 0.001; ^****^ *p* < 0.0001; NS, not significant. (J) MMP13 and HMGB1 fluorescence intensity (n = 6 per group). ^*^ *p* < 0.05; ^**^ *p* < 0.01; ^***^ *p* < 0.001; ****p < 0.0001; NS, not significant. Each point represents one individual mouse. (K) Representative MIF/Cd79a and HMGB1/Cd79a immunofluorescence images from Sham, OA, OA + hydrogel, and OA + SQH knee joints at 24 weeks. (L) MIF fluorescence intensity (n = 6 per group). ^*^ *p* < 0.05; ^**^ *p* < 0.01; ^***^ *p* < 0.001; ^****^ *p* < 0.0001; NS, not significant. (M) HMGB1‐positive B cells (n = 6 per group). ^*^ *p* < 0.05; ^**^ *p* < 0.01; ^***^ *p* < 0.001; ^****^ *p* < 0.0001; NS, not significant.

The therapeutic efficacy of SQH was then evaluated in a DMM‐induced OA mouse model. Mice received intra‐articular injections of hydrogel alone or SQH on postoperative days 14, 28, and 42, corresponding to three injections at two‐week intervals, and knee joints were harvested for terminal analysis at 24 weeks after DMM surgery (Figure 7G). H&E and Safranin O/Fast Green staining showed severe cartilage erosion and matrix loss in OA and hydrogel‐only groups, whereas SQH treatment preserved cartilage structure and Safranin O staining (Figure 7H). Consistently, terminal OARSI histological scores at 24 weeks were significantly lower in the SQH group than in the hydrogel‐only group, indicating reduced histological OA severity at the examined endpoint (Figure 7I).

Immunofluorescence staining further showed reduced MMP13 expression and restored HMGB1 signal in SQH‐treated OA joints, supporting decreased cartilage degeneration and senescence‐associated changes (Figure 7H,J). In addition, MIF immunofluorescence was higher in OA and hydrogel‐only joints but was reduced in SQH‐treated joints at the 24‐week endpoint (Figure 7K,L). The proportion of HMGB1‐positive B cells was also increased in SQH‐treated joints compared with OA and hydrogel‐only groups (Figure 7K,M), consistent with attenuation of senescence‐associated B‐cell alterations at the 24‐week endpoint. Together, these findings show that SQH treatment was associated with attenuation of senescence‐associated B‐cell alterations, reduced MIF immunofluorescence, and decreased cartilage degeneration.

## 3 Discussion

In this study, we systematically profiled cellular and molecular alterations in knee joints from OA and aged mice, with a focus on B cells and MIF signaling. Single‐cell transcriptomics revealed substantial remodeling of the joint microenvironment, including a reduction in B‐cell abundance and marked alterations in structural‐cell compartments. Notably, senescence‐associated B‐cell remodeling was accompanied by increased MIF abundance and enhanced computationally predicted MIF‐associated communication with chondrocytes in OA and aged joints. This immune‐structural cell crosstalk was associated with pro‐inflammatory and catabolic changes in chondrocytes, disruption of cartilage extracellular‐matrix homeostasis, and accelerated cartilage degradation. Genetic deletion and antibody‐mediated blockade of MIF mitigated these pathological changes, supporting a functional role for MIF in OA progression. Based on these findings, we engineered a dual‐action SQH hydrogel that combines senolytic quercetin with MIF‐siRNA‐loaded microspheres. This approach attenuated chondrocyte degeneration, reduced cellular senescence, and helped restore a more homeostatic B cell–chondrocyte signaling environment.

B cells are central components of adaptive immunity, producing antibodies and regulating immune responses [47]. Senescent or dysfunctional B cells are commonly characterized by cell‐cycle arrest, reduced metabolic and proliferative capacity, pro‐inflammatory mediator production, and imbalanced B‐cell subset composition [48, 49]. Such alterations have been implicated in degenerative and inflammatory diseases [50, 51]. In OA, however, the contribution of B cells remains incompletely understood. Our data suggest that B cells in OA undergo senescence‐associated remodeling, reflected by reduced abundance, altered transcriptional states, and impaired proliferative programs within joint tissues. In addition, the increased proportion of plasma cell‐related subpopulations in OA is consistent with previous reports of altered B‐cell expansion and plasma‐cell differentiation in OA [14].This differentiation bias may arise from persistent stimulation within the inflammatory joint microenvironment, where cartilage‐degradation products and cytokines such as IL‐6 and TNF‐α can activate B‐cell receptors or Toll‐like receptors and promote plasma‐cell development [52, 53, 54]. Antibody secretion and pro‐inflammatory cytokine release may further intensify local inflammation, disrupt cartilage matrix homeostasis, and accelerate chondrocyte dysfunction. These observations position senescence‐associated B‐cell remodeling as a potential immune driver of OA pathology.

Our study supports a role for MIF in OA‐associated cartilage degeneration, although its effects appear to be context dependent. Previous studies have reported divergent findings regarding MIF in OA. Rowe et al. showed that *Mif* deletion reduced naturally occurring age‐related OA in older mice, whereas neither *Mif* deletion nor systemic MIF neutralization produced comparable protection in a DMM model using young adult mice [24]. In contrast, we observed reduced DMM‐associated cartilage pathology in constitutive *Mif^−/−^* mice under the conditions used in the present study. Differences in animal age, sex, genetic background, DMM procedure, experimental endpoint, and histological scoring may contribute to these apparently different outcomes. These findings therefore suggest that the contribution of MIF to OA may vary according to disease context and experimental setting rather than representing a uniform effect across models. Interestingly, the same study also reported increased MIF secretion by human OA chondrocytes compared with normal chondrocytes, suggesting that the contribution of MIF may depend on disease context and local cellular sources. In contrast, Liu et al. reported lower circulating MIF protein levels and reduced MIF mRNA expression in articular cartilage from patients with end‐stage OA and therefore proposed a potentially protective role for MIF [25]. However, this conclusion was primarily based on cross‐sectional associations rather than direct functional manipulation of MIF, and cartilage MIF expression was not correlated with circulating MIF levels in that study. These observations suggest that circulating MIF, bulk cartilage MIF expression, and locally produced MIF within specific cellular compartments may represent biologically distinct aspects of MIF signaling. From this perspective, our identification of enhanced MIF production in senescence‐associated B cells and the MIF‐dependent effects of OA‐ and aging‐associated B cells on chondrocytes provides a cell‐resolved mechanistic framework that may help reconcile these apparently divergent findings. Mechanistically, MIF produced by senescence‐associated B cells may bind the CD74‐CD44 receptor complex on chondrocytes and activate downstream inflammatory pathways, including NF‐κB signaling. This may increase pro‐inflammatory cytokines such as IL‐1β and IL‐6 and matrix‐degrading enzymes such as MMP13 and MMP9 [55, 56], thereby promoting a senescence‐associated secretory phenotype and accelerating matrix degradation. In parallel, MIF may enhance oxidative stress through NADPH oxidase activation and mitochondrial dysfunction, increasing reactive oxygen species and DNA‐damage responses [57, 58]. Excessive ROS can reinforce MIF secretion and NF‐κB activation, creating a self‐amplifying loop that aggravates inflammation, mitochondrial stress, and chondrocyte senescence [59]. Thus, targeting the MIF‐NF‐κB‐ROS axis may represent a rational strategy for interrupting OA‐associated inflammatory senescence.

The interaction between MIF and B cells may also involve autocrine and paracrine feedback. B cells can produce MIF and express MIF receptors, enabling reciprocal amplification of MIF signaling [60]. Previous studies have shown that MIF promotes B‐cell survival through the CD74‐CD44 receptor complex and NF‐κB activation [61]. In inflammatory arthritis, NF‐κB signaling sustains B‐cell survival and promotes pro‐inflammatory cytokine secretion, contributing to synovial inflammation and cartilage injury [62, 63]. These observations support a dual‐action therapeutic strategy that simultaneously suppresses upstream B‐cell senescence and downstream MIF signaling. Consistent with emerging multifunctional biomaterial strategies for local modulation of the osteoarthritic joint microenvironment [64], the present study used an SQH hydrogel combining quercetin with MIF‐siRNA‐loaded PLGA microspheres for local composite delivery. The PLGA microspheres showed gradual siMIF release over the measured 96‐h in vitro interval, while quercetin may modulate senescence‐associated B‐cell alterations and associated inflammatory responses. In vivo, SQH treatment was associated with reduced MIF immunofluorescence at the terminal endpoint. By attenuating B‐cell senescence, limiting excessive plasma‐cell differentiation, and inhibiting MIF–CD74/CD44‐NF‐κB signaling, SQH hydrogel may reduce inflammatory cytokine and matrix‐degrading enzyme production, preserve cartilage homeostasis, and delay OA progression.

In conclusion, our findings identify senescence‐associated B cells as key mediators of OA through MIF‐driven chondrocyte degeneration. Neutralizing MIF or suppressing MIF signaling attenuates joint inflammation, reduces B‐cell senescence, and preserves cartilage integrity. The SQH provided a dual‐action therapeutic strategy by attenuating senescence‐associated B‐cell alterations and suppressing MIF signaling within the joint, thereby reducing chondrocyte degeneration and OA progression.

Future studies should aim to unravel the complex intercellular signaling networks orchestrated by other immune cells and cytokines in the osteoarthritic context.

## 4 Materials and Methods

### 4.1 Experimental Design

This study integrated single‐cell RNA sequencing (scRNA‐seq), spatial transcriptomics, tissue‐level validation, cell‐based functional assays, genetic MIF deficiency, and a biomaterial intervention to define the role of the senescence‐associated B cell–MIF axis in osteoarthritis (OA) progression and joint aging. The discovery cohort comprised 24 mouse knee‐joint samples from healthy controls, naturally aged mice (15 and 30 months), and destabilization of the medial meniscus (DMM)‐induced OA mice collected 2, 4, 8, 12, or 24 weeks after surgery. Major joint‐cell populations and B‐cell states were characterized by scRNA‐seq; MIF‐centered cell–cell communication was evaluated computationally and spatially; and mechanistic relevance was tested in MIF‐deficient mice, joint‐derived B cell–chondrocyte co‐cultures, MIF‐neutralization and recombinant‐MIF add‐back experiments, and treatment with a siMIF microsphere/quercetin hydrogel (SQH).

Animals were allocated to experimental groups before intervention, and tissue processing, image acquisition, and outcome assessment were performed using matched protocols. Histological scoring and quantitative image analysis were conducted by investigators blinded to group identity. For all in vivo, tissue‐based, and sample‐level single‐cell transcriptomic comparisons, the individual mouse was defined as the biological experimental unit. Multiple sections, regions of interest, microscopic fields, or cells obtained from the same mouse were treated as technical or nested observations and were summarized within each mouse before between‐group statistical analysis. Thus, each displayed data point represents one mouse unless otherwise specified. Technical replicates were not treated as independent biological replicates. For biological‐replicate‐level assessment, cell‐level senescence AUCell scores were summarized by calculating the mean score for B cells from each individual mouse.

### 4.2 Single‐Cell Library Preparation, Sequencing, and Preprocessing

Single‐cell 3′ gene‐expression libraries were generated using the 10x Genomics Chromium Single Cell 3′ Gene Expression platform. Libraries were sequenced on an Illumina platform using paired‐end sequencing. Raw FASTQ files were processed using Cell Ranger (version 5.0.0) and aligned to the mouse GRCm38/mm10 reference genome to generate gene–barcode expression matrices. Each mouse was processed as an independent biological sample, and sample identity was retained throughout quality control and multi‐sample integration. Ambient/background RNA contamination was corrected using CellBender before downstream analysis. Quality control was performed separately for each sample based on UMI counts, the number of detected genes, and the proportions of mitochondrial and ribosomal transcripts. Cells with an erythrocyte‐associated transcript fraction >1% were excluded, and the maximum mitochondrial‐transcript threshold was capped at 25%. Predicted doublets were identified and removed using Scrublet. After quality control, the top 2,000 highly variable genes were identified for each sample. Principal‐component analysis was performed, and Harmony was used for multi‐sample integration and batch‐effect correction. The first 20 principal components were retained for downstream clustering. Graph‐based clustering was performed using the Louvain algorithm, and the integrated data were visualized using t‐SNE and UMAP.

### 4.3 Preparation of Single‐Cell Suspension

Under sterile conditions, the knee joint tissues of mice were obtained fresh with sterile surgical scissors. The tissues were washed twice with RPMI 1640 cold medium plus 0.04% bovine serum albumin (BSA). Subsequently, the tissue was cut into 0.5 mm^3^ fragments with surgical scissors and cultured in a freshly prepared enzymatic digestion solution containing 1 mg/ml collagenase IV in DMEM supplemented with 5% fetal bovine serum (FBS). In a constant temperature incubator, digest at 37°C for 30–60 min, and gently reverse every 5–10 min to promote dissociation. After digestion, the cell suspension was passed through a 40 µm BD cell filter once or twice to remove debris and undigested tissue. The filtered suspension was centrifuged at 300 × g for 5 min at 4°C. The obtained cell particles were resuspended in an appropriate volume of medium and mixed with red blood cell lysis buffer (MACS, Catalog No. 130‐094‐183) 1:1. The mixture was incubated at 4°C for 10 min to lyse red blood cells. The cells were centrifuged at 300 × g for 5 min and the supernatant was discarded. The pellets were washed with medium once and centrifuged again under the same conditions to remove the supernatant. Finally, the cell particles were resuspended in 100 µL medium, and the cell concentration and viability were evaluated by a Luna automatic cell counter.

### 4.4 Cell Clustering and Cell‐Type Annotation

Cluster‐enriched marker genes used for cell‐type annotation were identified using the FindMarkers function in Seurat with the Wilcoxon rank‐sum test. Genes with log‐fold change > 0.25 and expression in at least 20% of cells within the corresponding cluster were considered representative cluster markers. These cell‐level marker analyses were used for descriptive cluster annotation and were not treated as biological‐replicate‐level differential‐expression analyses. Normalised enrichment scores were z‑scaled across clusters and visualised with ComplexHeatmap (v2.14.0). Cell type annotation was performed using the SingleR (version 1.4.1) [65] with the MouseRNAseqData reference dataset, in conjunction with cluster‐specific marker‐gene expression and literature‐curated lineage markers. SingleR‐based assignments were cross‐checked against marker‐gene expression patterns, and a complementary SCINA‐based semi‐supervised annotation was used to assess annotation consistency. For clusters in which a definitive lineage identity was not sufficiently supported by the combined annotation evidence, neutral transcriptional labels were assigned.

### 4.5 Mouse‐Level Pseudobulk Differential‐Expression Analysis

For cross‐condition differential‐expression analysis of B cells, the individual mouse was treated as the biological experimental unit. Raw UMI counts from B cells were extracted from the unintegrated RNA assay and aggregated separately for each mouse by summing gene counts across all B cells derived from that animal, thereby generating one pseudobulk expression profile per mouse. Harmony‐corrected expression values were used for dimensionality reduction and visualization but were not used for pseudobulk differential‐expression testing. Pseudobulk count matrices were analyzed using edgeR. Lowly expressed genes were removed using filterByExpr, and library‐size differences were normalized using the trimmed mean of M‐values (TMM) method. A negative‐binomial generalized linear model was fitted with experimental group as the explanatory variable, and differential expression was assessed using the quasi‐likelihood framework. Comparisons between each OA or aging group and the Control group were performed using prespecified contrasts. The three individual mice within each group were retained as independent biological replicates throughout the analysis.

P values were adjusted for multiple testing using the Benjamini–Hochberg procedure. Genes with an adjusted *p* value < 0.05 and an absolute fold change > 1.5 were considered differentially expressed. Mouse‐level pseudobulk differential‐expression results were used as the input for downstream cross‐condition enrichment analyses.

Significant pseudobulk DEGs from each prespecified OA‐ or aging‐versus‐Control contrast were used for GO and KEGG over‐representation analyses. The gene universe was defined as all genes retained after pseudobulk expression filtering and tested in the corresponding differential‐expression contrast. GO and KEGG enrichment analyses were performed using clusterProfiler, with the Gene Ontology (GO; release v2021.0918) and Kyoto Encyclopedia of Genes and Genomes (KEGG; release v2021.09) databases. P values from enrichment analyses were adjusted for multiple testing using the Benjamini–Hochberg procedure, and adjusted p < 0.05 was considered significant. For preranked GSEA, all genes tested in the corresponding pseudobulk contrast were ranked according to log2‐fold change. Positive normalized enrichment scores (NES) indicate enrichment toward the OA or aging group, whereas negative NES values indicate enrichment toward the Control group. GSEA *p* values were adjusted using the Benjamini–Hochberg procedure.

### 4.6 AUCell‐Based Senescence‐Associated Transcriptional Scoring

A senescence‐associated gene set comprising nine genes (Cdkn1a, Cdkn2a, Cdkn2b, Trp53, Rb1, Gadd45a, Gadd45b, Btg2, and Glb1) was used for AUCell scoring. The RNA assay of the B‐cell Seurat object was used, and the raw count matrix was extracted using GetAssayData. Genes absent from the expression matrix were excluded before scoring. Gene‐expression rankings were generated for each cell using AUCell_buildRankings, and AUCell scores were calculated using AUCell_calcAUC, with aucMaxRank set to ceiling(0.05 × N), where N denotes the total number of genes in the expression matrix. A random seed of 123 was used for reproducibility, and other AUCell parameters were left at their default settings. The resulting AUCell score was treated as a continuous measure, with higher scores indicating greater enrichment of the predefined senescence‐associated transcriptional gene set. No binary cutoff was used to classify individual B cells as senescent or non‐senescent.

### 4.7 Cell‐Cell Interaction Analysis

Cell–cell ligand–receptor interaction analysis was performed using the CellChat [66] R package (version 1.1.3). A normalized expression matrix was first imported, and a CellChat object was created using the createCellChat function. Preprocessing was conducted with default parameters using the identifyOverExpressedGenes, identifyOverExpressedInteractions, and projectData functions. Potential ligand–receptor interactions were then inferred using the computeCommunProb, filterCommunication, and computeCommunProbPathway functions. Finally, the overall cell–cell communication network was aggregated using the aggregateNet function.

### 4.8 Pseudotime Trajectory Analysis

B cells were subjected to pseudotime trajectory analysis using Monocle (version 2.14.0). Cells were organized into a CellDataSet object and ordered along an inferred transcriptional trajectory using the standard Monocle workflow. Dimensionality reduction and trajectory reconstruction were performed using the DDRTree algorithm with default parameters. State 2 was designated as the reference root based primarily on the expression patterns of the proliferation‐ and cell‐cycle‐associated genes Ccna2, Mki67, Pcna, and Top2a, which were consistent with a relatively proliferative transcriptional state. Hmgb1 expression was additionally examined as a state‐associated feature along the inferred trajectory. Cells were subsequently ordered in pseudotime relative to this reference root. The resulting trajectory was interpreted as an inferred transcriptional topology rather than as direct evidence of an in vivo lineage transition.

### 4.9 Spatial Transcriptomic Visualization

Spatial transcriptomics was performed on one control knee‐joint section and one OA‐24 W knee‐joint section, each derived from an independent mouse (biological n = 1 per condition). Accordingly, these spatial datasets were used to provide representative anatomical context and to support, rather than independently establish, group‐level differences between control and OA joints.

Spatial transcriptomic data from control and OA‐24 W mouse knee joints were processed and normalized using a common workflow. Normalized gene‐expression values were mapped to their corresponding spatial coordinates and overlaid on the tissue images. Spatial feature plots were generated for Cd79a and MIF. Spots with detectable Cd79a expression were operationally defined as B‐cell‐associated spots for spatial visualization. Because individual Visium spots may contain multiple cell types, this designation was used to indicate candidate regions containing a B‐cell‐associated transcriptional signal rather than a pure B‐cell population or a cell‐specific source of MIF.

Spatially resolved ligand–receptor interactions were analyzed using stLearn (version 1.2.2) and its spatially constrained ligand–receptor permutation framework. Literature‐curated mouse ligand–receptor pairs were obtained from the connectomeDB2020_lit database using species = “mouse”. Spot‐level ligand–receptor scores were calculated using st.tl.cci.run with min_spots = 20, distance = None, n_pairs = 10,000, and random_state = 0. With distance = None, each spatial neighborhood comprised the focal Visium spot and its immediately adjacent spots.

For each ligand–receptor pair, the observed spatial score at each spot was compared with a background distribution generated from 10,000 non‐interacting gene pairs selected to have expression distributions comparable to those of the corresponding ligand and receptor genes. P values were adjusted using the Benjamini–Hochberg method with st.tl.cci.adj_pvals, setting correct_axis = “spot”, adj_method = “fdr_bh”, and pval_adj_cutoff = 0.05. Spots with adjusted *p* < 0.05 were considered significant ligand–receptor hotspots. These adjusted *p* values reflect spot‐level spatial testing within the analyzed section and were not interpreted as evidence of a biologically replicated difference between Control and OA conditions.

Significant MIF–Cd74 and MIF–Cd44 interaction hotspots were mapped onto the corresponding tissue sections and examined in relation to Cd79a‐positive B‐cell‐associated spots and spatial senescence‐associated scores. Identical color scales and plotting parameters were applied to the control and OA‐24 W sections.

### 4.10 Cell Expansion

Primary mouse chondrocytes were isolated from articular cartilage obtained from Control or OA mice, according to the experimental design. Chondrocytes were cultured and proliferated in Dulbecco's Modified Eagle Medium (DMEM supplied by Life Technologies in China), which was enriched with 10% fetal bovine serum (FBS sourced from HyClone, a part of Thermo Scientific in the USA), 1% combined antibiotic–antimycotic solution (containing 100 units/mL penicillin, 100 µg/mL streptomycin, and 0.25 µg/mL amphotericin B), and 1 mM sodium pyruvate (also from Life Technologies, China). The chondrocyte cultures were maintained at optimal conditions of 37°C, 5% carbon dioxide, and 95% relative humidity until they reached the third passage. Nutrient media were refreshed at intervals of two to three days. When cell growth reached 80–90% confluence, cells were detached using trypsin (provided by Life Technologies, China). This enzymatic treatment was consistently employed for both subculturing and collecting cells throughout the experimental process.

### 4.11 Joint‐Derived B Cell Isolation and Magnetic Bead Sorting

Mouse knee joint tissues were collected under sterile conditions from Control, OA‐8 W, OA‐24 W, *Mif^−/−^*, and 15‐month‐old aging mice. Joint‐associated tissues were minced into small fragments and enzymatically digested to obtain single‐cell suspensions as described above. Briefly, tissues were digested in DMEM containing collagenase IV at 37°C with gentle agitation, followed by filtration through a 40 µm cell strainer to remove undigested tissue debris. After centrifugation, red blood cells were lysed using red blood cell lysis buffer, and the remaining cells were washed and resuspended in magnetic‐activated cell sorting buffer.

B cells were isolated from joint‐derived single‐cell suspensions by positive magnetic selection using CD19 MicroBeads, mouse (Miltenyi Biotec B.V. & Co. KG, Bergisch Gladbach, Germany; cat. no. 130‐121‐301). Cell pellets were resuspended in 90 µL of MACS buffer per 1 × 10^7^ total cells, and 10 µL of CD19 MicroBeads was added per 1 × 10^7^cells. Following incubation at 4°C for 10 min, the cells were washed and resuspended in MACS buffer. The labeled cell suspension was applied to an LS Column (Miltenyi Biotec; cat. no. 130‐042‐401) placed in a MidiMACS Separator (Miltenyi Biotec; cat. no. 130‐042‐302). The LS Column was pre‐equilibrated with 3 mL of MACS buffer before sample loading. After loading, the column was washed with 3 mL of MACS buffer. The column was then removed from the separator, and the retained CD19^+^ cells were eluted with 5 mL of MACS buffer using the supplied plunger. Post‐sort B‐cell purity and viability were assessed by flow cytometry. CD19‐positive B‐cell purity exceeded 95%, and cell viability exceeded 90% before subsequent experiments.

Sorted joint‐derived CD19^+^ B cells from each group were used for senescence validation, MIF secretion analysis, and Transwell co‐culture experiments with primary chondrocytes. *Mif^−/−^* B cells were isolated from MIF knockout mice using the same procedure and served as MIF‐deficient B cells in loss‐of‐function and recombinant MIF add‐back experiments.

For each experimental group, B cells were independently isolated from six male donor mice (n = 6 biological replicates per group). Cells obtained from different donor mice were processed separately and were not pooled; thus, each biological replicate represented an independent B‐cell isolation from one individual mouse. Primary chondrocytes used for the co‐culture experiments were isolated from healthy control mice and used at passage 3. Technical wells and microscopic fields derived from the same B‐cell preparation were not treated as independent replicates; measurements were aggregated within each preparation to generate one biological‐replicate‐level value for statistical analysis.

### 4.12 Transwell Co‐Culture and MIF Rescue/Add‐Back Treatments

Primary mouse chondrocytes at passage 3 were seeded onto sterile coverslips in the lower chambers of 24‐well Transwell plates at a density of 5 × 10^4^ cells per well and cultured overnight to allow adherence. Joint‐derived CD19^+^ B cells isolated from Control, OA‐8 W, OA‐24 W, *Mif^−/−^*, and 15‐month‐old aging mice were then added to the upper chambers of 0.4‐µm pore size Transwell inserts at a density of 1 × 10^5^ cells per insert, corresponding to a B cell:chondrocyte ratio of 2:1. The co‐culture was maintained in complete DMEM containing 10% fetal bovine serum and 1% penicillin–streptomycin at 37°C in a humidified atmosphere with 5% CO_2_ for 48 h.

For the comparison of B cells from different sources, chondrocytes were co‐cultured with CD19^+^ B cells from Control, OA‐8 W, OA‐24 W, *Mif^−/−^*, or 15‐month‐old aging mice under identical conditions. After co‐culture, chondrocytes in the lower chambers were fixed for immunofluorescence staining of COL2A1, FN1, HMGB1, MMP13, P16, and P21, or subjected to SA‐β‐gal staining to assess cellular senescence.

For MIF blockade experiments, OA‐24W‐derived CD19^+^ B cells were co‐cultured with primary chondrocytes in the presence of an anti‐MIF neutralizing antibody at 10 µg/mL. An isotype‐matched IgG antibody at the same concentration was used as the control. For MIF add‐back experiments, *Mif^−/−^* CD19^+^ B cells were co‐cultured with primary chondrocytes in the presence of recombinant mouse MIF at 10 ng/mL. The anti‐MIF antibody, IgG control, or recombinant MIF was added at the beginning of co‐culture and maintained throughout the 48‐h treatment period. After treatment, chondrocytes were analyzed for senescence‐associated markers, including P16, P21, and SA‐β‐gal staining. All experimental groups were processed in parallel using the same number of chondrocytes and B cells. Immunofluorescence images were acquired under identical imaging settings within each experiment, and fluorescence intensity or positive‐cell percentage was quantified using ImageJ.

### 4.13 Quantification of MIF Secretion by ELISA

Sorted joint‐derived CD19^+^ B cells from Control, OA‐8 W, OA‐24 W, *Mif^−/−^*, and 15‐month‐old aging mice were seeded in 24‐well plates at a density of 1×10^5^ cells per well in 500 µL of complete culture medium. Cells were cultured at 37°C in a humidified atmosphere containing 5% CO_2_ for 48 h. Cell‐free wells containing the same volume of complete culture medium were included as background controls. At the end of the incubation period, culture supernatants were collected and centrifuged at 300 × g for 5 min to remove residual cells and large cellular debris. The clarified supernatants were transferred to fresh tubes, aliquoted, and stored at −80°C until analysis. Repeated freeze–thaw cycles were avoided.

MIF concentrations in B‐cell‐conditioned media were quantified using a Mouse MIF ELISA Kit (Macklin, Shanghai, China; cat. no. M709406; 48‐test format) according to the manufacturer's instructions. Standards, samples, and medium blanks were added to the ELISA plate and processed using the incubation, washing, detection‐antibody, enzyme‐conjugate, substrate‐development, and reaction‐termination procedures specified by the manufacturer. Samples and standards were analyzed in technical duplicate. Absorbance was measured at 450 nm using a microplate reader. After subtraction of the blank absorbance, MIF concentrations were calculated from the standard curve using a four‐parameter logistic regression model. Samples with absorbance values outside the valid standard‐curve range were diluted with the sample diluent supplied with the kit and reanalyzed.

MIF secretion was initially expressed as the concentration measured in the culture supernatant (pg/mL). Where viable cell numbers were determined at the end of the 48‐h culture period, the total amount of MIF released per well was calculated by multiplying the measured concentration by the culture volume and was normalized to the number of viable cells, with the results expressed as pg per 1×10^5^ viable B cells. Supernatants from MIF^−/−^ B cells were analyzed in parallel as MIF‐deficient controls.

### 4.14 SA‐β‐gal Staining and Quantification

SA‐β‐gal staining was performed using a senescence β‐galactosidase staining kit according to the manufacturer's instructions (Beyotime Biotechnology, Shanghai, China; cat. no. C0602). For B‐cell staining, sorted CD19^+^ B cells from each group were seeded onto poly‐L‐lysine‐coated slides or culture plates before fixation. For chondrocyte staining, primary chondrocytes were stained after Transwell co‐culture with the indicated B‐cell groups or after MIF rescue/add‐back treatments.

Briefly, cells were washed twice with PBS and fixed with the provided fixation solution for 15 min at room temperature. After washing, cells were incubated with freshly prepared SA‐β‐gal staining solution at pH 6.0 overnight at 37°C in a dry incubator without CO_2_. After staining, cells were washed with PBS and imaged under a bright‐field microscope. SA‐β‐gal‐positive cells were identified by blue staining.

For quantification, at least five randomly selected fields were analyzed for each sample. The number of SA‐β‐gal‐positive cells and the total number of cells were counted using ImageJ. The percentage of SA‐β‐gal‐positive cells was calculated as:

$$\begin{array}{cc} & \mathrm{SA}-\beta -\mathrm{gal}-\mathrm{positive}\;\mathrm{cells}\;\left(\%\right)\\ & =\mathrm{SA}-\beta -\mathrm{gal}-\mathrm{positive}\;\mathrm{cells}/\mathrm{total}\;\mathrm{cells}\times 100\% \end{array}$$

All images within the same experiment were acquired under identical microscope settings, and quantification was performed in a blinded manner.

### 4.15 Fabrication of the siMIF Microsphere/Quercetin Hydrogel (SQH)

The composite carrier hydrogel was prepared with hyaluronic acid (HA, 3 mg/mL; 0.3% w/v), gelatin (35 mg/mL; 3.5% w/v), and fibrinogen (20 mg/mL; 2% w/v). To prepare the quercetin‐containing hydrogel, quercetin was added to the hydrogel solution at a final concentration of 50 µM (15.112 µg/mL), followed by sonication for 30 min. For MIF‐siRNA delivery, MIF‐targeting siRNA (sense, 5′‐GCCUAUGUUCAUCGUGAAC‐dTdT‐3′; antisense, 5′‐GUUCACGAUGAACAUAGGC‐dTdT‐3′) was encapsulated in poly(lactic‐co‐glycolic acid) (PLGA) microspheres with a 50:50 lactide:glycolide ratio. The siRNA‐loaded microspheres or corresponding empty microspheres were subsequently incorporated into the quercetin‐containing hydrogel. In the final SQH formulation, the encapsulated MIF‐siRNA concentration was 100 µg/mL. The dimensions of the fabricated PLGA microspheres were characterized by scanning electron microscopy (SEM). For the in vitro release assay, Cy3‐labeled MIF‐siRNA‐loaded PLGA microspheres were incubated in PBS at 37°C under static conditions before incorporation into the final SQH hydrogel. At predetermined time points over 96 h, the microspheres were centrifuged at 2500 rpm for 5 min, and the supernatant was collected for fluorescence measurement. Released Cy3‐labeled MIF‐siRNA was quantified using a fluorescence microplate reader with excitation and emission wavelengths of 550 and 570 nm, respectively. The cumulative release experiment was independently performed using 12 separately prepared microsphere batches (n = 12). Measurements obtained from the same batch at different time points were treated as serial measurements rather than independent replicates. Rheological properties of the SQH were evaluated using a rheometer (TA Instruments, USA). Temperature‐sweep measurements were performed on the pre‐crosslinked SQH formulation from 15°C to 45°C at a heating rate of 1°C/min, with a constant frequency of 1 Hz and strain of 1%. Frequency‐sweep measurements were performed on the final UV‐crosslinked SQH at 17°C over an angular‐frequency range of 0.1–10 rad/s at a constant strain of 1%. Both rheological measurements were performed using 12 independently prepared SQH samples (n = 12). Measurements collected from the same hydrogel sample across different temperatures or frequencies were treated as serial measurements rather than independent replicates. These rheological profiles were used for descriptive material characterization. To assess the distribution of the microspheres within the fabricated hydrogel, siMIF PLGA µs containing rhodamine were introduced into the hydrogel matrix and observed under a fluorescent microscope. Post‐integration, the spread of the rhodamine‐tagged PLGA µs in the hydrogel was examined via a confocal microscope. Chondrocytes were co‐cultured with different hydrogels and observed under fluorescent microscopes. Fluorescence imaging was used to examine the spatial association of Cy3‐labeled siMIF microsphere‐derived signals with phalloidin‐stained chondrocytes under the imaging conditions used. MIF immunofluorescence staining was performed in treated chondrocytes to assess changes in MIF protein signal. Phalloidin (Thermo Fisher Scientific, USA) was used to stain the F‐actin for 1 h and incubated with DAPI (Thermo Fisher Scientific, USA) to stain the nucleus for 5 min in turn.

### 4.16 Intra‐Articular Administration of SQH

For in vivo treatment, 10 µL of freshly prepared SQH containing 0.151 µg quercetin, 0.20 mg PLGA microspheres, and a total encapsulated siMIF payload of 1.0 µg was administered into the operated knee joint by intra‐articular injection on days 14, 28, and 42 after DMM surgery, corresponding to three injections at two‐week intervals. Hydrogel‐only control animals received the same injection volume of an otherwise identical carrier formulation without quercetin or PLGA microspheres on the same schedule.

### 4.17 Histological Analysis and Immunofluorescence Staining

As in our previous research [67], tissues were fixed in 10% neutral buffered formalin for a minimum of 72 h, followed by decalcification in 10% EDTA (BioShop) for 14 to 21 days. After decalcification, samples were embedded in paraffin for further processing. Serial sagittal sections (4 µm thick) were cut through the center of the most severely affected osteoarthritic region and stained with hematoxylin and eosin (H&E) and Safranin O (Saf‐O) following standard protocols. Histological evaluation of the knee joint sections was conducted by two independent, blinded observers using the OARSI (Osteoarthritis Research Society International) grading system. For each section, the scores assigned by the two blinded observers were averaged to obtain a section‐level OARSI score. The section‐level scores from multiple sections of the same mouse were then averaged to generate one mouse‐level OARSI score. Thus, each mouse contributed one independent OARSI value to the statistical analysis.

For immunofluorescence staining, tissues were initially fixed in 4% paraformaldehyde (PFA) and permeabilized with PBS containing 0.5% Triton X‐100 for 20 min. This was followed by a blocking step with 3% bovine serum albumin (BSA) containing 0.025% Triton X‐100 and 5% fetal bovine serum (FBS) at room temperature for 30 min. Samples were then incubated with primary antibodies overnight at 4°C. After three washes with PBS, tissues were incubated with appropriate secondary antibodies. A second round of PBS washes was performed, followed by nuclear counterstaining with DAPI for 5 min. Immunofluorescence images were captured using a confocal microscope (Zeiss, Germany). To maximize the use of limited tissue resources and achieve multi‐target colocalization within the same spatial coordinate framework, we applied a sequential immunofluorescence strategy to selected paraffin‐embedded sections.

### 4.18 Immunofluorescence Image Acquisition and Quantitative Analysis

Immunofluorescence images used for quantitative comparisons were acquired under identical microscope settings within each experiment, including laser intensity, detector gain, magnification, and image‐acquisition parameters. Sections processed without primary antibody were used as negative controls to assess nonspecific secondary‐antibody fluorescence. Quantitative image analysis was performed using ImageJ by investigators blinded to group identity. Anatomical regions of interest (ROIs), including the synovium, subchondral bone, bone marrow, and articular cartilage, were defined according to the corresponding analysis.

For tissue‐based quantitative analyses, three sections per mouse were analyzed, with three non‐overlapping microscopic fields acquired from each predefined anatomical region per section. Fluorescence thresholds were determined based on the corresponding negative‐control sections and were applied consistently to all images within the same experiment. For fluorescence‐intensity measurements, signal intensity was quantified within the predefined ROI after background exclusion. Field‐level measurements were first averaged within each section, and the three section‐level values were subsequently averaged to generate one mouse‐level value for statistical analysis. Thus, each mouse contributed one independent biological value.

For colocalization‐based cell‐percentage measurements, MIF‐positive B cells and HMGB1‐positive B cells were defined as MIF/Cd79a or HMGB1/Cd79a double‐positive cells, respectively, and were expressed relative to the total number of Cd79a‐positive B cells within the analyzed ROI. HMGB1‐positive chondrocytes were expressed as the number of HMGB1‐positive chondrocytes divided by the total number of chondrocytes within the articular‐cartilage ROI.

### 4.19 Animals

*Mif* ^−/−^ mice and wild‐type C57BL/6J were purchased from Jin Pharmaceutical Technology Co., Ltd. (Nanjing, China). *Mif* ^+/−^ heterozygous mice were generated by crossing *Mif* ^−/−^ mice with wild‐type C57BL/6J mice. All experiments were approved by the Animal Committee of the First Affiliated Hospital of Nanjing Medical University (No. IACUC‐2303016).

Male WT and *Mif^−/−^* littermate mice on a C57BL/6J background were used for the DMM experiments at 8 weeks of age. Genotypes were confirmed by PCR genotyping before experimental allocation. Mice of different genotypes were co‐housed under the same specific‐pathogen‐free conditions. Animals were randomly allocated to experimental groups before surgery, and no animals were excluded after allocation. Independent terminal cohorts were used for the 8‐ and 24‐week DMM endpoints. For OARSI analysis, 9 mice per genotype were analyzed at each terminal time point. Separate cohorts of 6 mice per group were used for the immunofluorescence analyses shown in Figure 4H,K.

### 4.20 Construction of Mice Model of OA

We established a mouse model of knee osteoarthritis using the DMM method (Destabilization of the Medial Meniscus). Mice were anesthetized, and a medial incision was made at the knee joint to expose the joint capsule. The medial meniscotibial ligament (MMTL) was then transected to induce joint instability. Control‐operated mice underwent the same surgical procedure except for the transection of the meniscal ligament and were used as controls. Intra‐articular injection of SQH hydrogel (OA+SQH hydrogel group) or hydrogel only (OA+hydrogel only group) was performed as treatment in the mice in the OA+hydrogel only group and the OA+SQH hydrogel group. After the surgery, the mice were allowed to freely move in separate cages and were provided with standard food and water. Twenty‐four weeks later, the mice were euthanized for further study.

### 4.21 Statistical Analysis

Statistical analyses were performed using SPSS software (version 19.0, SPSS Inc., Chicago, IL, USA), GraphPad Prism (version 8.0, GraphPad Software Inc., San Diego, CA, USA), and R where appropriate. Unless otherwise specified, data are presented as mean ± SD, and all statistical tests were two‐sided. For in vivo and tissue‐based analyses, the individual mouse was considered the biological experimental unit. Measurements obtained from multiple sections, regions of interest, microscopic fields, or technical replicates from the same mouse were summarized within each mouse before group‐level statistical analysis. For comparisons involving two independent groups, an appropriate two‐sided test was used according to the characteristics of the endpoint and variance structure. For multigroup comparisons, an appropriate omnibus test or model was first performed, followed, where applicable, by prespecified or post hoc pairwise contrasts with multiplicity adjustment. For Figure 4F, the 8‐ and 24‐week groups comprised independent terminal cohorts and were not treated as repeated measurements. OARSI scores were analyzed as ordinal outcomes using a cumulative‐link model including genotype, terminal time, and their interaction, followed by multiplicity‐adjusted comparisons between WT and Mif^−/−^ mice at each terminal time point. For Figure 4K, genotype, disease condition, and their interaction were evaluated using a two‐factor analysis, followed by adjusted simple comparisons where appropriate. OARSI scores assigned by the two blinded observers were first averaged within each section, and section‐level scores from the same mouse were subsequently averaged to generate one mouse‐level OARSI value. For the four‐group OARSI analysis in Figure 7I, mouse‐level OARSI scores were analyzed using a Jeffreys‐prior penalized cumulative‐link ordinal logistic model with treatment group as the explanatory variable, followed by six prespecified pairwise contrasts with Holm adjustment for multiple testing. For microsphere characterization, the independently prepared microsphere batch was considered the experimental unit. Individual particles measured within the same batch were treated as subsamples rather than independent replicates. Cumulative‐release and rheological profiles were treated as descriptive serial measurements; repeated measurements obtained from the same independently prepared sample across time, temperature, or frequency were not treated as independent replicates. Complete panel‐specific statistical information, including sample size, data‐summary definition, statistical test or model, sidedness, prespecified or post hoc contrasts, multiplicity adjustment, effect estimates, 95% confidence intervals, and exact adjusted P values, is provided in Table S3. A two‐sided adjusted *p* < 0.05 was considered statistically significant where multiplicity adjustment was applied. NS denotes not statistically significant.

## Author Contributions

Y.S. and K.D. conceived and supervised the study. W.L., Y.W., Y.Y., Y.C., J.L., X.M., and Q.Z. performed the experiments and analyzed the data. Q.W., Z.Z., and Z.L. contributed to data collection, data interpretation, and technical support. W.L. and Y.W. drafted the manuscript. All authors critically reviewed and revised the manuscript and approved the final version for submission.

## Funding

This study was supported by the National Natural Science Foundation of China (Grant Number: 82472423, 82172446, 82202660, and 82402819).

## Conflicts of Interest

The authors declare no conflicts of interest.

## Supporting information




**Supporting File**: advs78543‐sup‐0001‐SuppMat.docx.

## Data Availability

The data that support the findings of this study are available from the corresponding author upon reasonable request.
