# Supplementary material for: Integrated Single‐Cell and Spatial Transcriptomics Reveal Convergence on a Senescence‐Associated B Cell–MIF Signaling Axis in Joint Aging and Osteoarthritis Progression
Source: Adv Sci (Weinh). 2026 Oct 29:e78543. Online ahead of print. doi: 10.1002/advs.78543 (PMC13720388; doi:10.1002/advs.78543)
Supplement: Supplementary file 1 [file ADVS-9999-e78543-s001.docx]

**Supplementary Table S1. Complete gene set used for AUCell-based senescence-associated transcriptional scoring**

The senescence-associated gene set contained nine genes, which were grouped into three biological categories according to their reported functional associations with cell-cycle arrest, p53/DNA-damage responses, and senescence-associated cellular phenotypes. All nine genes were scored together as a single gene set using AUCell; the three categories were used only for biological organization and interpretation. No additional genes were included in the AUCell gene set.

| **Biological category** | **Complete gene set** | **Rationale for inclusion** | **Reference** |
| --- | --- | --- | --- |
| Cell-cycle arrest | Cdkn1a, Cdkn2a, Cdkn2b, Rb1 | Captures stable cell-cycle arrest, a core feature of cellular senescence. CDKN1A/p21 and CDKN2A/p16 converge on RB-dependent cell-cycle control, while CDKN2B/p15 can also promote RB-dependent senescence in appropriate cellular contexts. | Refs. S1-S3 |
| p53/DNA-damage response | Trp53, Gadd45a, Gadd45b | Captures p53-linked DNA-damage and stress-response features associated with senescence. GADD45 family proteins participate in DNA-damage responses, cell-cycle control, and modulation of senescence, with context-dependent effects. | Refs. S1, S4 |
| Senescence-associated phenotype | Btg2, Glb1 | Captures senescence-associated antiproliferative and lysosomal features. BTG2 contributes to maintenance of replicative senescence, whereas GLB1 encodes the lysosomal beta-galactosidase responsible for senescence-associated beta-galactosidase activity. | Refs. S5, S6 |

**Supporting references**

S1. Gorgoulis V, Adams PD, Alimonti A, Bennett DC, Bischof O, Bishop C, Campisi J, Collado M, Evangelou K, Ferbeyre G, Gil J, Hara E, Krizhanovsky V, Jurk D, Maier AB, Narita M, Niedernhofer L, Passos JF, Robbins PD, Schmitt CA, Sedivy J, Vougas K, von Zglinicki T, Zhou D, Serrano M, Demaria M. Cellular Senescence: Defining a Path Forward. Cell. 2019 Oct 31;179(4):813-827. doi: 10.1016/j.cell.2019.10.005. PMID: 31675495.

S2. Hernandez-Segura A, Nehme J, Demaria M. Hallmarks of Cellular Senescence. Trends Cell Biol. 2018 Jun;28(6):436-453. doi: 10.1016/j.tcb.2018.02.001. Epub 2018 Feb 21. PMID: 29477613.

S3. Fuxe J, Akusjärvi G, Goike HM, Roos G, Collins VP, Pettersson RF. Adenovirus-mediated overexpression of p15INK4B inhibits human glioma cell growth, induces replicative senescence, and inhibits telomerase activity similarly to p16INK4A. Cell Growth Differ. 2000 Jul;11(7):373-84. PMID: 10939591.

S4. Zaidi MR, Liebermann DA. Gadd45 in Senescence. Adv Exp Med Biol. 2022;1360:109-116. doi: 10.1007/978-3-030-94804-7_8. PMID: 35505166; PMCID: PMC9902168.

S5. Wheaton K, Muir J, Ma W, Benchimol S. BTG2 antagonizes Pin1 in response to mitogens and telomere disruption during replicative senescence. Aging Cell. 2010 Oct;9(5):747-60. doi: 10.1111/j.1474-9726.2010.00601.x. Epub 2010 Aug 4. PMID: 20569234.

S6. Lee BY, Han JA, Im JS, Morrone A, Johung K, Goodwin EC, Kleijer WJ, DiMaio D, Hwang ES. Senescence-associated beta-galactosidase is lysosomal beta-galactosidase. Aging Cell. 2006 Apr;5(2):187-95. doi: 10.1111/j.1474-9726.2006.00199.x. PMID: 16626397.

**Supplementary Table S2. Annotation of B-cell transcriptional clusters**

| **Cluster** | **Assigned B-cell subtype/state** | **Marker gene** | **Reference** |
| --- | --- | --- | --- |
| Cluster 1 | Immature B cells | Dnajc7; Atp1b1; Rag1 | Ref. 37 (Lee et al., 2021) |
| Cluster 2 | Naive B cells | H2-Aa; H2-Eb1; H2-Ab1 | Ref. 38 (Daamen et al., 2023) |
| Cluster 3 | Memory B cells | Ms4a1; Ifi30 | Ref. 39 (van Langelaar et al., 2020) |
| Cluster 4 | Germinal-center dark-zone B cells (GC DZ B) | Hist1h1b; Hist1h2ap; Hist1h2ae | Refs. 40-42 (Holmes et al., 2020; Espinoza et al., 2023; Morgan & Tergaonkar, 2022) |
| Cluster 5 | Pro-B cells | Vpreb1; Igll1; Dntt | Refs. 42-43 (Morgan & Tergaonkar, 2022; Rothenberg, 2014) |
| Cluster 6 | Germinal-center dark-zone B cells (GC DZ B) | Ube2c; Cenpf; Hmgb2 | Refs. 40-42 (Holmes et al., 2020; Espinoza et al., 2023; Morgan & Tergaonkar, 2022) |
| Cluster 7 | Germinal-center dark-zone B cells (GC DZ B) | H2afx; Mki67 | Refs. 40-42 (Holmes et al., 2020; Espinoza et al., 2023; Morgan & Tergaonkar, 2022) |
| Cluster 8 | Undefined B cells | Ccnd2; Il4i1; Dut | No subtype-specific reference assigned in the current manuscript |
| Cluster 9 | Marginal-zone B cells (MZ B) | Dnajc7; H2-Aa; H2-Eb1 | Ref. 44 (Mabbott & Gray, 2014) |
| Cluster 10 | Plasma cells | Jchain; Slpi; Mt1 | Ref. 45 (Xu et al., 2020) |
| Cluster 11 | Pro-B cells | Rag1; Dntt | Refs. 42-43 (Morgan & Tergaonkar, 2022; Rothenberg, 2014) |
| Cluster 12 | Cycling pre-B cells | Hist1h1b; Ube2c; Hmgb2 | Ref. 37 (Lee et al., 2021) |
| Cluster 13 | Interferon-induced B cells (IFN-induced B) | Ifit3;Slfn5;Isg15 | Ref. 47 (Khsheibun et al., 2014) |
| Cluster 14 | Top2a-expressing B cells (Top2A-B) | H2afx; Mki67; Cenpf | Ref. 42 (Morgan & Tergaonkar, 2022) |
| Cluster 15 | Plasmablasts | Il4i1; Hbb-bt; Cenpf | Ref. 46 (Tarte et al., 2003) |

**Supporting references**

37. Lee RD, Munro SA, Knutson TP, LaRue RS, Heltemes-Harris LM, Farrar MA. Single-cell analysis identifies dynamic gene expression networks that govern B cell development and transformation. Nat Commun. 2021 Nov 25;12(1):6843. doi: 10.1038/s41467-021-27232-5. PMID: 34824268; PMCID: PMC8617197.

38. Daamen AR, Alajoleen RM, Grammer AC, Luo XM, Lipsky PE. Single-cell RNA sequencing analysis reveals the heterogeneity of IL-10 producing regulatory B cells in lupus-prone mice. Front Immunol. 2023 Dec 14;14:1282770. doi: 10.3389/fimmu.2023.1282770. PMID: 38155972; PMCID: PMC10752970.

39. van Langelaar J, Rijvers L, Smolders J, van Luijn MM. B and T Cells Driving Multiple Sclerosis: Identity, Mechanisms and Potential Triggers. Front Immunol. 2020 May 8;11:760. doi: 10.3389/fimmu.2020.00760. PMID: 32457742; PMCID: PMC7225320.

40. Holmes AB, Corinaldesi C, Shen Q, Kumar R, Compagno N, Wang Z, Nitzan M, Grunstein E, Pasqualucci L, Dalla-Favera R, Basso K. Single-cell analysis of germinal-center B cells informs on lymphoma cell of origin and outcome. J Exp Med. 2020 Oct 5;217(10):e20200483. doi: 10.1084/jem.20200483. PMID: 32603407; PMCID: PMC7537389.

41. Espinoza DA, Le Coz C, Cruz Cabrera E, Romberg N, Bar-Or A, Li R. Distinct stage-specific transcriptional states of B cells derived from human tonsillar tissue. JCI Insight. 2023 Apr 10;8(7):e155199. doi: 10.1172/jci.insight.155199. PMID: 36809296; PMCID: PMC10132144.

42. Morgan D, Tergaonkar V. Unraveling B cell trajectories at single cell resolution. Trends Immunol. 2022 Mar;43(3):210-229. doi: 10.1016/j.it.2022.01.003. Epub 2022 Jan 25. PMID: 35090788.

43. Rothenberg EV. Transcriptional control of early T and B cell developmental choices. Annu Rev Immunol. 2014;32:283-321. doi: 10.1146/annurev-immunol-032712-100024. Epub 2014 Jan 22. PMID: 24471430; PMCID: PMC3994230.

44. Mabbott NA, Gray D. Identification of co-expressed gene signatures in mouse B1, marginal zone and B2 B-cell populations. Immunology. 2014 Jan;141(1):79-95. doi: 10.1111/imm.12171. PMID: 24032749; PMCID: PMC3893852.

45. Xu AQ, Barbosa RR, Calado DP. Genetic timestamping of plasma cells in vivo reveals tissue-specific homeostatic population turnover. Elife. 2020 Nov 2;9:e59850. doi: 10.7554/eLife.59850. PMID: 33136000; PMCID: PMC7682985.

46. Tarte K, Zhan F, De Vos J, Klein B, Shaughnessy J Jr. Gene expression profiling of plasma cells and plasmablasts: toward a better understanding of the late stages of B-cell differentiation. Blood. 2003 Jul 15;102(2):592-600. doi: 10.1182/blood-2002-10-3161. Epub 2003 Mar 27. PMID: 12663452.

47. Khsheibun R, Paperna T, Volkowich A, Lejbkowicz I, Avidan N, Miller A. Gene expression profiling of the response to interferon beta in Epstein-Barr-transformed and primary B cells of patients with multiple sclerosis. PloS one. 2014;9(7):e102331.

 **Supplementary Fig. S1. Per-mouse t-SNE distribution of B-cell subtypes during OA progression and joint aging.**

t-SNE plots of joint B cells are shown separately for each individual scRNA-seq sample. Each panel represents one mouse, and cells are colored according to the annotated B-cell subtype/state. The plots illustrate the distribution of B-cell subtypes across individual animals and allow visual assessment of inter-mouse consistency across the Control, OA-2W, OA-4W, OA-8W, OA-12W, OA-24W, 15M, and 30M groups, with three biological replicates per group.

**Supplementary Fig.S2** **Per-mouse composition of B-cell subtypes during OA progression and joint aging.**

Stacked bar plots show the relative proportions of the annotated B-cell subtypes within the B-cell compartment of each individual scRNA-seq sample. Each bar represents one individual mouse. For each mouse, the proportion of each subtype was calculated as the number of cells assigned to that subtype/state divided by the total number of B cells recovered from the same mouse after quality control. Control, OA-2W, OA-4W, OA-8W, OA-12W, OA-24W, 15M, and 30M groups each contained three biological replicates.

**Supplementary Table S3. Panel-Specific Statistical Information**

| **Figure panel** | **Endpoint** | **n/group** | **Summary / error bars** | **Statistical test / model** | **Sidedness** | **Planned contrast** | **Multiplicity adjustment** | **Effect estimate (Group A− Group B)** | **95% CI** | **Exact adjusted P value** | **Significance** |
| --- | --- | --- | --- | --- | --- | --- | --- | --- | --- | --- | --- |
| Fig. 1G | B-cell proportion | 3 mice/group | Raw proportions; mean ± SD | One-way ANOVA on logit-transformed B-cell proportions | Two-sided | 30M vs Control | Holm adjustment across 7 prespecified Control comparisons | −2.9840 | −3.1860 to −2.7821 | 6.075 × 10⁻15 | **** |
| Fig. 1G | B-cell proportion | 3 mice/group | Raw proportions; mean ± SD | One-way ANOVA on logit-transformed B-cell proportions | Two-sided | 15M vs Control | Holm adjustment across 7 prespecified Control comparisons | −1.8252 | −2.0271 to −1.6232 | 5.555 × 10⁻12 | **** |
| Fig. 1G | B-cell proportion | 3 mice/group | Raw proportions; mean ± SD | One-way ANOVA on logit-transformed B-cell proportions | Two-sided | OA-2W vs Control | Holm adjustment across 7 prespecified Control comparisons | −1.4618 | −1.6637 to −1.2598 | 5.427 × 10⁻11 | **** |
| Fig. 1G | B-cell proportion | 3 mice/group | Raw proportions; mean ± SD | One-way ANOVA on logit-transformed B-cell proportions | Two-sided | OA-4W vs Control | Holm adjustment across 7 prespecified Control comparisons | −2.2091 | −2.4110 to −2.0072 | 5.497 × 10⁻13 | **** |
| Fig. 1G | B-cell proportion | 3 mice/group | Raw proportions; mean ± SD | One-way ANOVA on logit-transformed B-cell proportions | Two-sided | OA-8W vs Control | Holm adjustment across 7 prespecified Control comparisons | −2.0472 | −2.2492 to −1.8453 | 1.259 × 10⁻12 | **** |
| Fig. 1G | B-cell proportion | 3 mice/group | Raw proportions; mean ± SD | One-way ANOVA on logit-transformed B-cell proportions | Two-sided | OA-12W vs Control | Holm adjustment across 7 prespecified Control comparisons | −1.7182 | −1.9202 to −1.5163 | 9.345 × 10⁻12 | **** |
| Fig. 1G | B-cell proportion | 3 mice/group | Raw proportions; mean ± SD | One-way ANOVA on logit-transformed B-cell proportions | Two-sided | OA-24W vs Control | Holm adjustment across 7 prespecified Control comparisons | −2.2165 | −2.4185 to −2.0146 | 5.497 × 10⁻13 | **** |
| Fig. 1I | Cd79a-positive area, ST | 9 mice/group | Raw values; mean ± SD | Compartment-specific one-way ANOVA | Two-sided | 30M vs Control | Holm adjustment across 9 prespecified comparisons | -1.9533 | -2.7138 to -1.1929 | 0.000116407 | *** |
| Fig. 1I | Cd79a-positive area, ST | 9 mice/group | Raw values; mean ± SD | Compartment-specific one-way ANOVA | Two-sided | OA-24W vs Control | Holm adjustment across 9 prespecified comparisons | -1.5300 | -2.2904 to -0.7696 | 0.001432231 | ** |
| Fig. 1I | Cd79a-positive area, ST | 9 mice/group | Raw values; mean ± SD | Compartment-specific one-way ANOVA | Two-sided | 30M vs OA-24W | Holm adjustment across 9 prespecified comparisons | -0.4233 | -1.1838 to 0.3371 | 0.523773294 | NS |
| Fig. 1I | Cd79a-positive area, SB | 9 mice/group | Raw values; mean ± SD | Compartment-specific one-way ANOVA | Two-sided | 30M vs Control | Holm adjustment across 9 prespecified comparisons | -5.0089 | -6.5724 to -3.4454 | 5.403741 × 10^-6 | **** |
| Fig. 1I | Cd79a-positive area, SB | 9 mice/group | Raw values; mean ± SD | Compartment-specific one-way ANOVA | Two-sided | OA-24W vs Control | Holm adjustment across 9 prespecified comparisons | -3.8667 | -5.4302 to -2.3032 | 0.000159639 | *** |
| Fig. 1I | Cd79a-positive area, SB | 9 mice/group | Raw values; mean ± SD | Compartment-specific one-way ANOVA | Two-sided | 30M vs OA-24W | Holm adjustment across 9 prespecified comparisons | -1.1422 | -2.7057 to 0.4213 | 0.433974202 | NS |
| Fig. 1I | Cd79a-positive area, BM | 9 mice/group | Raw values; mean ± SD | Compartment-specific one-way ANOVA | Two-sided | 30M vs Control | Holm adjustment across 9 prespecified comparisons | -1.2444 | -1.5563 to -0.9326 | 1.500102 × 10^-7 | **** |
| Fig. 1I | Cd79a-positive area, BM | 9 mice/group | Raw values; mean ± SD | Compartment-specific one-way ANOVA | Two-sided | OA-24W vs Control | Holm adjustment across 9 prespecified comparisons | -1.3456 | -1.6574 to -1.0337 | 4.039045 × 10^-8 | **** |
| Fig. 1I | Cd79a-positive area, BM | 9 mice/group | Raw values; mean ± SD | Compartment-specific one-way ANOVA | Two-sided | 30M vs OA-24W | Holm adjustment across 9 prespecified comparisons | 0.1011 | -0.2107 to 0.4129 | 0.523773294 | NS |
| Fig. 2O | MMP13 fluorescence intensity | 9 mice/group | Raw values; mean ± SD | Welch ANOVA + Games-Howell | Two-sided | 30M vs Control | Games-Howell adjustment across 3 pairwise comparisons | 1.1389 | 0.6467 to 1.6310 | 0.000425 | *** |
| Fig. 2O | MMP13 fluorescence intensity | 9 mice/group | Raw values; mean ± SD | Welch ANOVA + Games-Howell | Two-sided | 30M vs OA-24W | Games-Howell adjustment across 3 pairwise comparisons | -1.0878 | -2.1530 to -0.0226 | 0.045298 | * |
| Fig. 2O | MMP13 fluorescence intensity | 9 mice/group | Raw values; mean ± SD | Welch ANOVA + Games-Howell | Two-sided | Control vs OA-24W | Games-Howell adjustment across 3 pairwise comparisons | -2.2267 | -3.2489 to -1.2044 | 0.000648 | *** |
| Fig. 2P | HMGB1-positive B cells (%) | 9 mice/group | Raw values; mean ± SD | One-way ANOVA + Tukey HSD | Two-sided | 30M vs Control | Tukey HSD adjustment across 3 pairwise comparisons | -18.2389 | -22.3650 to -14.1127 | 2.041 × 10^-10 | **** |
| Fig. 2P | HMGB1-positive B cells (%) | 9 mice/group | Raw values; mean ± SD | One-way ANOVA + Tukey HSD | Two-sided | 30M vs OA-24W | Tukey HSD adjustment across 3 pairwise comparisons | -1.3000 | -5.4261 to 2.8261 | 0.714558 | NS |
| Fig. 2P | HMGB1-positive B cells (%) | 9 mice/group | Raw values; mean ± SD | One-way ANOVA + Tukey HSD | Two-sided | Control vs OA-24W | Tukey HSD adjustment across 3 pairwise comparisons | 16.9389 | 12.8127 to 21.0650 | 8.918 × 10^-10 | **** |
| Fig. 2Q | HMGB1-positive chondrocytes (%) | 9 mice/group | Raw values; mean ± SD | One-way ANOVA + Tukey HSD | Two-sided | 30M vs Control | Tukey HSD adjustment across 3 pairwise comparisons | -25.9089 | -30.0445 to -21.7733 | 1.270 × 10^-13 | **** |
| Fig. 2Q | HMGB1-positive chondrocytes (%) | 9 mice/group | Raw values; mean ± SD | One-way ANOVA + Tukey HSD | Two-sided | 30M vs OA-24W | Tukey HSD adjustment across 3 pairwise comparisons | -1.5156 | -5.6511 to 2.6200 | 0.636169 | NS |
| Fig. 2Q | HMGB1-positive chondrocytes (%) | 9 mice/group | Raw values; mean ± SD | One-way ANOVA + Tukey HSD | Two-sided | Control vs OA-24W | Tukey HSD adjustment across 3 pairwise comparisons | 24.3933 | 20.2578 to 28.5289 | 4.766 × 10^-13 | **** |
| Fig. 3E | MIF fluorescence intensity | 6 mice/group | Raw values; mean ± SD | Welch ANOVA + Games-Howell | Two-sided | 30M vs Control | Games-Howell adjustment across 3 pairwise comparisons | 0.9200 | 0.5530 to 1.2870 | 0.001014 | ** |
| Fig. 3E | MIF fluorescence intensity | 6 mice/group | Raw values; mean ± SD | Welch ANOVA + Games-Howell | Two-sided | OA-24W vs Control | Games-Howell adjustment across 3 pairwise comparisons | 1.5933 | 0.8744 to 2.3122 | 0.001853 | ** |
| Fig. 3E | MIF fluorescence intensity | 6 mice/group | Raw values; mean ± SD | Welch ANOVA + Games-Howell | Two-sided | 30M vs OA-24W | Games-Howell adjustment across 3 pairwise comparisons | -0.6733 | -1.3938 to 0.0471 | 0.065436 | NS |
| Fig. 3F | MIF-positive B cells (%) | 6 mice/group | Raw values; mean ± SD | One-way ANOVA + Tukey HSD | Two-sided | 30M vs Control | Tukey HSD adjustment across 3 pairwise comparisons | 11.3850 | 5.2768 to 17.4932 | 0.000595 | *** |
| Fig. 3F | MIF-positive B cells (%) | 6 mice/group | Raw values; mean ± SD | One-way ANOVA + Tukey HSD | Two-sided | OA-24W vs Control | Tukey HSD adjustment across 3 pairwise comparisons | 19.2417 | 13.1334 to 25.3499 | 1.856 × 10^-6 | **** |
| Fig. 3F | MIF-positive B cells (%) | 6 mice/group | Raw values; mean ± SD | One-way ANOVA + Tukey HSD | Two-sided | 30M vs OA-24W | Tukey HSD adjustment across 3 pairwise comparisons | -7.8567 | -13.9649 to -1.7484 | 0.011699 | * |
| Fig. 3G | Serum MIF concentration | 6 mice/group | Raw values; mean ± SD | One-way ANOVA + Tukey HSD | Two-sided | 30M vs Control | Tukey HSD adjustment across 3 pairwise comparisons | 184.4017 | 128.4973 to 240.3060 | 1.045 × 10^-6 | **** |
| Fig. 3G | Serum MIF concentration | 6 mice/group | Raw values; mean ± SD | One-way ANOVA + Tukey HSD | Two-sided | OA-24W vs Control | Tukey HSD adjustment across 3 pairwise comparisons | 255.3517 | 199.4473 to 311.2560 | 1.451 × 10^-8 | **** |
| Fig. 3G | Serum MIF concentration | 6 mice/group | Raw values; mean ± SD | One-way ANOVA + Tukey HSD | Two-sided | 30M vs OA-24W | Tukey HSD adjustment across 3 pairwise comparisons | -70.9500 | -126.8544 to -15.0456 | 0.012783 | * |
| Fig. 3H | Synovial-fluid MIF concentration | 6 mice/group | Raw values; mean ± SD | One-way ANOVA + Tukey HSD | Two-sided | 30M vs Control | Tukey HSD adjustment across 3 pairwise comparisons | 344.3767 | 249.0827 to 439.6706 | 3.264 × 10^-7 | **** |
| Fig. 3H | Synovial-fluid MIF concentration | 6 mice/group | Raw values; mean ± SD | One-way ANOVA + Tukey HSD | Two-sided | OA-24W vs Control | Tukey HSD adjustment across 3 pairwise comparisons | 453.5800 | 358.2861 to 548.8739 | 8.259 × 10^-9 | **** |
| Fig. 3H | Synovial-fluid MIF concentration | 6 mice/group | Raw values; mean ± SD | One-way ANOVA + Tukey HSD | Two-sided | 30M vs OA-24W | Tukey HSD adjustment across 3 pairwise comparisons | -109.2033 | -204.4973 to -13.9094 | 0.024070 | * |
| Fig. 4B | Body weight (g) | 6 mice/group | Raw values; mean ± SD | Unpaired Student's t test | Two-sided | Mif-/- vs WT | Not applicable; single prespecified comparison | -3.8183 | -6.2581 to -1.3786 | 0.005851 | ** |
| Fig. 4C | Body length (cm) | 6 mice/group | Raw values; mean ± SD | Unpaired Student's t test | Two-sided | Mif-/- vs WT | Not applicable; single prespecified comparison | -0.2000 | -0.3656 to -0.0344 | 0.022646 | * |
| Fig. 4D | Serum MIF concentration (ng/mL) | 6 mice/group | Raw values; mean ± SD | Welch ANOVA + Games-Howell | Two-sided | WT vs Mif+/- | Games-Howell adjustment across 3 pairwise comparisons | 30.4567 | -13.2924 to 74.2057 | 0.185228 | NS |
| Fig. 4D | Serum MIF concentration (ng/mL) | 6 mice/group | Raw values; mean ± SD | Welch ANOVA + Games-Howell | Two-sided | WT vs Mif-/- | Games-Howell adjustment across 3 pairwise comparisons | 201.5733 | 168.7763 to 234.3704 | 8.188 × 10^-6 | **** |
| Fig. 4D | Serum MIF concentration (ng/mL) | 6 mice/group | Raw values; mean ± SD | Welch ANOVA + Games-Howell | Two-sided | Mif+/- vs Mif-/- | Games-Howell adjustment across 3 pairwise comparisons | 171.1167 | 131.5834 to 210.6499 | 5.865 × 10^-5 | **** |
| Fig. 4F | Terminal OARSI histological score | 9 mice/genotype at each terminal time point | Raw mouse-level scores; mean ± SD | Cumulative-link ordinal logistic model | Two-sided | Genotype × terminal-time interaction | Not applicable to interaction term | Interaction proportional-odds ratio = 0.2696 | 0.0150 to 4.8562 | 0.374156 | NS |
| Fig. 4F | Terminal OARSI histological score | 9 mice/genotype at each terminal time point | Raw mouse-level scores; mean ± SD | Cumulative-link ordinal model; simple genotype contrast | Two-sided | Mif-/- vs WT at 8W | Holm adjustment across 2 prespecified genotype contrasts | Proportional-odds ratio = 0.1095 | 0.0166 to 0.7208 | 0.021418 | * |
| Fig. 4F | Terminal OARSI histological score | 9 mice/genotype at each terminal time point | Raw mouse-level scores; mean ± SD | Cumulative-link ordinal model; simple genotype contrast | Two-sided | Mif-/- vs WT at 24W | Holm adjustment across 2 prespecified genotype contrasts | Proportional-odds ratio = 0.02952 | 0.00321 to 0.2713 | 0.003710 | ** |
| Fig. 4H | MIF fluorescence intensity | 6 mice/group | Raw values; mean ± SD | Welch t test | Two-sided | Mif-/- vs WT | Not applicable; single prespecified comparison for this endpoint | -1.0517 | -1.3191 to -0.7843 | 0.000145 | *** |
| Fig. 4H | MMP13 fluorescence intensity | 6 mice/group | Raw values; mean ± SD | Unpaired Student's t test | Two-sided | Mif-/- vs WT | Not applicable; single prespecified comparison for this endpoint | -0.8450 | -1.1434 to -0.5466 | 8.791 × 10^-5 | **** |
| Fig. 4K | MIF-positive B cells (%) | 6 mice/genotype-condition group | Raw values; mean ± SD | Two-factor linear model with HC3 robust SE | Two-sided | Genotype × disease-condition interaction | Not applicable to interaction term | Difference-in-differences = -13.1000 | -18.6565 to -7.5435 | 8.303 × 10^-5 | **** |
| Fig. 4K | MIF-positive B cells (%) | 6 mice/genotype-condition group | Raw values; mean ± SD | Two-factor linear model with HC3 robust SE; simple contrast | Two-sided | Mif-/- Control vs WT Control | Holm adjustment across 2 prespecified genotype contrasts | -10.9000 | -14.0122 to -7.7878 | 4.601 × 10^-7 | **** |
| Fig. 4K | MIF-positive B cells (%) | 6 mice/genotype-condition group | Raw values; mean ± SD | Two-factor linear model with HC3 robust SE; simple contrast | Two-sided | Mif-/- OA-24W vs WT OA-24W | Holm adjustment across 2 prespecified genotype contrasts | -24.0000 | -28.6031 to -19.3969 | 1.514 × 10^-9 | **** |
| Fig. 4K | HMGB1-positive B cells (%) | 6 mice/genotype-condition group | Raw values; mean ± SD | Two-way ANOVA | Two-sided | Genotype × disease-condition interaction | Not applicable to interaction term | Difference-in-differences = 2.9500 | -2.9887 to 8.8887 | 0.312485 | NS |
| Fig. 4K | HMGB1-positive B cells (%) | 6 mice/genotype-condition group | Raw values; mean ± SD | Two-way ANOVA; simple contrast | Two-sided | Mif-/- Control vs WT Control | Holm adjustment across 2 prespecified genotype contrasts | 5.4833 | 1.2840 to 9.6826 | 0.013078 | * |
| Fig. 4K | HMGB1-positive B cells (%) | 6 mice/genotype-condition group | Raw values; mean ± SD | Two-way ANOVA; simple contrast | Two-sided | Mif-/- OA-24W vs WT OA-24W | Holm adjustment across 2 prespecified genotype contrasts | 8.4333 | 4.2340 to 12.6326 | 0.000904 | *** |
| Fig. 5C | B-cell P21 fluorescence intensity | 6 B-cell donor mice/group | Raw values; mean ± SD | Welch ANOVA + prespecified Welch contrasts | Two-sided | OA-8W vs Control | Holm adjustment across 4 prespecified comparisons | 2.0983 | 1.6525 to 2.5442 | 4.376 × 10^-5 | **** |
| Fig. 5C | B-cell P21 fluorescence intensity | 6 B-cell donor mice/group | Raw values; mean ± SD | Welch ANOVA + prespecified Welch contrasts | Two-sided | OA-24W vs Control | Holm adjustment across 4 prespecified comparisons | 3.5600 | 2.9722 to 4.1478 | 4.335 × 10^-5 | **** |
| Fig. 5C | B-cell P21 fluorescence intensity | 6 B-cell donor mice/group | Raw values; mean ± SD | Welch ANOVA + prespecified Welch contrasts | Two-sided | 15M vs Control | Holm adjustment across 4 prespecified comparisons | 2.6517 | 2.2666 to 3.0367 | 1.893 × 10^-5 | **** |
| Fig. 5C | B-cell P21 fluorescence intensity | 6 B-cell donor mice/group | Raw values; mean ± SD | Welch ANOVA + prespecified Welch contrasts | Two-sided | OA-24W vs Mif-/- | Holm adjustment across 4 prespecified comparisons | 3.4867 | 2.8989 to 4.0744 | 4.335 × 10^-5 | **** |
| Fig. 5D | B-cell P16 fluorescence intensity | 6 B-cell donor mice/group | Raw values; mean ± SD | Welch ANOVA + prespecified Welch contrasts | Two-sided | OA-8W vs Control | Holm adjustment across 4 prespecified comparisons | 2.6300 | 2.0993 to 3.1607 | 5.436 × 10^-5 | **** |
| Fig. 5D | B-cell P16 fluorescence intensity | 6 B-cell donor mice/group | Raw values; mean ± SD | Welch ANOVA + prespecified Welch contrasts | Two-sided | OA-24W vs Control | Holm adjustment across 4 prespecified comparisons | 4.7300 | 3.9006 to 5.5594 | 5.436 × 10^-5 | **** |
| Fig. 5D | B-cell P16 fluorescence intensity | 6 B-cell donor mice/group | Raw values; mean ± SD | Welch ANOVA + prespecified Welch contrasts | Two-sided | 15M vs Control | Holm adjustment across 4 prespecified comparisons | 3.7100 | 3.1123 to 4.3077 | 5.247 × 10^-5 | **** |
| Fig. 5D | B-cell P16 fluorescence intensity | 6 B-cell donor mice/group | Raw values; mean ± SD | Welch ANOVA + prespecified Welch contrasts | Two-sided | OA-24W vs Mif-/- | Holm adjustment across 4 prespecified comparisons | 4.6100 | 3.7818 to 5.4382 | 5.436 × 10^-5 | **** |
| Fig. 5E | B-cell SA-beta-gal-positive cells (%) | 6 B-cell donor mice/group | Raw values; mean ± SD | One-way ANOVA + prespecified pooled-variance contrasts | Two-sided | OA-8W vs Control | Holm adjustment across 4 prespecified comparisons | 40.3683 | 32.1779 to 48.5588 | 2.368 × 10^-10 | **** |
| Fig. 5E | B-cell SA-beta-gal-positive cells (%) | 6 B-cell donor mice/group | Raw values; mean ± SD | One-way ANOVA + prespecified pooled-variance contrasts | Two-sided | OA-24W vs Control | Holm adjustment across 4 prespecified comparisons | 72.1017 | 63.9112 to 80.2921 | 2.701 × 10^-15 | **** |
| Fig. 5E | B-cell SA-beta-gal-positive cells (%) | 6 B-cell donor mice/group | Raw values; mean ± SD | One-way ANOVA + prespecified pooled-variance contrasts | Two-sided | 15M vs Control | Holm adjustment across 4 prespecified comparisons | 68.8500 | 60.6596 to 77.0404 | 4.193 × 10^-15 | **** |
| Fig. 5E | B-cell SA-beta-gal-positive cells (%) | 6 B-cell donor mice/group | Raw values; mean ± SD | One-way ANOVA + prespecified pooled-variance contrasts | Two-sided | OA-24W vs Mif-/- | Holm adjustment across 4 prespecified comparisons | 69.8800 | 61.6896 to 78.0704 | 4.193 × 10^-15 | **** |
| Fig. 5F | B-cell MIF secretion (pg/mL) | 6 B-cell donor mice/group | Raw values; mean ± SD | Welch ANOVA + prespecified Welch contrasts | Two-sided | OA-8W vs Control | Holm adjustment across 4 prespecified comparisons | 928.0 | 770.6850 to 1085.3150 | 2.150 × 10^-5 | **** |
| Fig. 5F | B-cell MIF secretion (pg/mL) | 6 B-cell donor mice/group | Raw values; mean ± SD | Welch ANOVA + prespecified Welch contrasts | Two-sided | OA-24W vs Control | Holm adjustment across 4 prespecified comparisons | 1625.1667 | 1398.1965 to 1852.1369 | 1.903 × 10^-5 | **** |
| Fig. 5F | B-cell MIF secretion (pg/mL) | 6 B-cell donor mice/group | Raw values; mean ± SD | Welch ANOVA + prespecified Welch contrasts | Two-sided | 15M vs Control | Holm adjustment across 4 prespecified comparisons | 1147.6667 | 917.6431 to 1377.6902 | 3.771 × 10^-5 | **** |
| Fig. 5F | B-cell MIF secretion (pg/mL) | 6 B-cell donor mice/group | Raw values; mean ± SD | Welch ANOVA + prespecified Welch contrasts | Two-sided | OA-24W vs Mif-/- | Holm adjustment across 4 prespecified comparisons | 1793.5000 | 1566.2192 to 2020.7808 | 1.903 × 10^-5 | **** |
| Fig. 5H | Chondrocyte FN1 fluorescence intensity | 6 B-cell donor mice/group | Raw values; mean ± SD | Welch ANOVA + prespecified Welch contrasts | Two-sided | OA-8W vs Control | Holm adjustment across 4 prespecified comparisons | 1.9000 | 1.5224 to 2.2776 | 1.147e-04 | *** |
| Fig. 5H | Chondrocyte FN1 fluorescence intensity | 6 B-cell donor mice/group | Raw values; mean ± SD | Welch ANOVA + prespecified Welch contrasts | Two-sided | OA-24W vs Control | Holm adjustment across 4 prespecified comparisons | 3.5500 | 2.8315 to 4.2685 | 1.147e-04 | *** |
| Fig. 5H | Chondrocyte FN1 fluorescence intensity | 6 B-cell donor mice/group | Raw values; mean ± SD | Welch ANOVA + prespecified Welch contrasts | Two-sided | 15M vs Control | Holm adjustment across 4 prespecified comparisons | 2.7500 | 2.2287 to 3.2713 | 1.147e-04 | *** |
| Fig. 5H | Chondrocyte FN1 fluorescence intensity | 6 B-cell donor mice/group | Raw values; mean ± SD | Welch ANOVA + prespecified Welch contrasts | Two-sided | OA-24W vs Mif-/- | Holm adjustment across 4 prespecified comparisons | 3.4500 | 2.7317 to 4.1683 | 1.147e-04 | *** |
| Fig. 5I | Chondrocyte COL2A1 fluorescence intensity | 6 B-cell donor mice/group | Raw values; mean ± SD | One-way ANOVA + prespecified pooled-variance contrasts | Two-sided | OA-8W vs Control | Holm adjustment across 4 prespecified comparisons | -0.6500 | -0.7250 to -0.5750 | 1.922e-15 | **** |
| Fig. 5I | Chondrocyte COL2A1 fluorescence intensity | 6 B-cell donor mice/group | Raw values; mean ± SD | One-way ANOVA + prespecified pooled-variance contrasts | Two-sided | OA-24W vs Control | Holm adjustment across 4 prespecified comparisons | -0.7883 | -0.8633 to -0.7134 | 4.102e-17 | **** |
| Fig. 5I | Chondrocyte COL2A1 fluorescence intensity | 6 B-cell donor mice/group | Raw values; mean ± SD | One-way ANOVA + prespecified pooled-variance contrasts | Two-sided | 15M vs Control | Holm adjustment across 4 prespecified comparisons | -0.5900 | -0.6650 to -0.5150 | 8.987e-15 | **** |
| Fig. 5I | Chondrocyte COL2A1 fluorescence intensity | 6 B-cell donor mice/group | Raw values; mean ± SD | One-way ANOVA + prespecified pooled-variance contrasts | Two-sided | OA-24W vs Mif-/- | Holm adjustment across 4 prespecified comparisons | -0.7367 | -0.8116 to -0.6617 | 1.534e-16 | **** |
| Fig. 5J | Chondrocyte HMGB1 fluorescence intensity | 6 B-cell donor mice/group | Raw values; mean ± SD | One-way ANOVA + prespecified pooled-variance contrasts | Two-sided | OA-8W vs Control | Holm adjustment across 4 prespecified comparisons | -0.5800 | -0.6589 to -0.5011 | 4.221e-14 | **** |
| Fig. 5J | Chondrocyte HMGB1 fluorescence intensity | 6 B-cell donor mice/group | Raw values; mean ± SD | One-way ANOVA + prespecified pooled-variance contrasts | Two-sided | OA-24W vs Control | Holm adjustment across 4 prespecified comparisons | -0.7300 | -0.8089 to -0.6511 | 8.378e-16 | **** |
| Fig. 5J | Chondrocyte HMGB1 fluorescence intensity | 6 B-cell donor mice/group | Raw values; mean ± SD | One-way ANOVA + prespecified pooled-variance contrasts | Two-sided | 15M vs Control | Holm adjustment across 4 prespecified comparisons | -0.6600 | -0.7389 to -0.5811 | 4.384e-15 | **** |
| Fig. 5J | Chondrocyte HMGB1 fluorescence intensity | 6 B-cell donor mice/group | Raw values; mean ± SD | One-way ANOVA + prespecified pooled-variance contrasts | Two-sided | OA-24W vs Mif-/- | Holm adjustment across 4 prespecified comparisons | -0.7100 | -0.7889 to -0.6311 | 1.204e-15 | **** |
| Fig. 5K | Chondrocyte MMP13 fluorescence intensity | 6 B-cell donor mice/group | Raw values; mean ± SD | One-way ANOVA + prespecified pooled-variance contrasts | Two-sided | OA-8W vs Control | Holm adjustment across 4 prespecified comparisons | 1.4133 | 1.0165 to 1.8102 | 1.100e-07 | **** |
| Fig. 5K | Chondrocyte MMP13 fluorescence intensity | 6 B-cell donor mice/group | Raw values; mean ± SD | One-way ANOVA + prespecified pooled-variance contrasts | Two-sided | OA-24W vs Control | Holm adjustment across 4 prespecified comparisons | 3.2983 | 2.9015 to 3.6952 | 1.024e-14 | **** |
| Fig. 5K | Chondrocyte MMP13 fluorescence intensity | 6 B-cell donor mice/group | Raw values; mean ± SD | One-way ANOVA + prespecified pooled-variance contrasts | Two-sided | 15M vs Control | Holm adjustment across 4 prespecified comparisons | 2.8700 | 2.4732 to 3.2668 | 1.229e-13 | **** |
| Fig. 5K | Chondrocyte MMP13 fluorescence intensity | 6 B-cell donor mice/group | Raw values; mean ± SD | One-way ANOVA + prespecified pooled-variance contrasts | Two-sided | OA-24W vs Mif-/- | Holm adjustment across 4 prespecified comparisons | 3.2150 | 2.8182 to 3.6118 | 1.386e-14 | **** |
| Fig. 5L | Chondrocyte P16 fluorescence intensity | 6 B-cell donor mice/group | Raw values; mean ± SD | Welch ANOVA + prespecified Welch contrasts | Two-sided | OA-8W vs Control | Holm adjustment across 4 prespecified comparisons | 1.7400 | 1.3395 to 2.1405 | 1.192e-04 | *** |
| Fig. 5L | Chondrocyte P16 fluorescence intensity | 6 B-cell donor mice/group | Raw values; mean ± SD | Welch ANOVA + prespecified Welch contrasts | Two-sided | OA-24W vs Control | Holm adjustment across 4 prespecified comparisons | 4.2900 | 3.5108 to 5.0692 | 1.065e-04 | *** |
| Fig. 5L | Chondrocyte P16 fluorescence intensity | 6 B-cell donor mice/group | Raw values; mean ± SD | Welch ANOVA + prespecified Welch contrasts | Two-sided | 15M vs Control | Holm adjustment across 4 prespecified comparisons | 2.3400 | 1.8124 to 2.8676 | 1.192e-04 | *** |
| Fig. 5L | Chondrocyte P16 fluorescence intensity | 6 B-cell donor mice/group | Raw values; mean ± SD | Welch ANOVA + prespecified Welch contrasts | Two-sided | OA-24W vs Mif-/- | Holm adjustment across 4 prespecified comparisons | 4.1700 | 3.3908 to 4.9492 | 1.065e-04 | *** |
| Fig. 5M | Chondrocyte P21 fluorescence intensity | 6 B-cell donor mice/group | Raw values; mean ± SD | One-way ANOVA + prespecified pooled-variance contrasts | Two-sided | OA-8W vs Control | Holm adjustment across 4 prespecified comparisons | 2.2500 | 1.6119 to 2.8881 | 1.309e-07 | **** |
| Fig. 5M | Chondrocyte P21 fluorescence intensity | 6 B-cell donor mice/group | Raw values; mean ± SD | One-way ANOVA + prespecified pooled-variance contrasts | Two-sided | OA-24W vs Control | Holm adjustment across 4 prespecified comparisons | 5.2500 | 4.6119 to 5.8881 | 1.296e-14 | **** |
| Fig. 5M | Chondrocyte P21 fluorescence intensity | 6 B-cell donor mice/group | Raw values; mean ± SD | One-way ANOVA + prespecified pooled-variance contrasts | Two-sided | 15M vs Control | Holm adjustment across 4 prespecified comparisons | 3.3800 | 2.7419 to 4.0181 | 1.077e-10 | **** |
| Fig. 5M | Chondrocyte P21 fluorescence intensity | 6 B-cell donor mice/group | Raw values; mean ± SD | One-way ANOVA + prespecified pooled-variance contrasts | Two-sided | OA-24W vs Mif-/- | Holm adjustment across 4 prespecified comparisons | 5.0700 | 4.4319 to 5.7081 | 2.170e-14 | **** |
| Fig. 5N | Chondrocyte SA-beta-gal-positive cells (%) | 6 B-cell donor mice/group | Raw values; mean ± SD | Welch ANOVA + prespecified Welch contrasts | Two-sided | OA-8W vs Control | Holm adjustment across 4 prespecified comparisons | 19.7417 | 15.5815 to 23.9018 | 1.149e-04 | *** |
| Fig. 5N | Chondrocyte SA-beta-gal-positive cells (%) | 6 B-cell donor mice/group | Raw values; mean ± SD | Welch ANOVA + prespecified Welch contrasts | Two-sided | OA-24W vs Control | Holm adjustment across 4 prespecified comparisons | 43.0700 | 34.0316 to 52.1084 | 1.149e-04 | *** |
| Fig. 5N | Chondrocyte SA-beta-gal-positive cells (%) | 6 B-cell donor mice/group | Raw values; mean ± SD | Welch ANOVA + prespecified Welch contrasts | Two-sided | 15M vs Control | Holm adjustment across 4 prespecified comparisons | 29.9883 | 24.2189 to 35.7578 | 1.149e-04 | *** |
| Fig. 5N | Chondrocyte SA-beta-gal-positive cells (%) | 6 B-cell donor mice/group | Raw values; mean ± SD | Welch ANOVA + prespecified Welch contrasts | Two-sided | OA-24W vs Mif-/- | Holm adjustment across 4 prespecified comparisons | 44.0500 | 35.0117 to 53.0883 | 1.149e-04 | *** |
| Fig. 5P | Chondrocyte P16 fluorescence intensity | 6 B-cell donor mice/group | Raw values; mean ± SD | Welch ANOVA + prespecified Welch contrasts | Two-sided | OA-24W+IgG vs Control | Holm adjustment across 5 prespecified comparisons | 3.7850 | 2.9350 to 4.6350 | 2.873e-04 | *** |
| Fig. 5P | Chondrocyte P16 fluorescence intensity | 6 B-cell donor mice/group | Raw values; mean ± SD | Welch ANOVA + prespecified Welch contrasts | Two-sided | OA-24W+anti-MIF vs Control | Holm adjustment across 5 prespecified comparisons | 0.0500 | -0.1119 to 0.2119 | 1.000000 | NS |
| Fig. 5P | Chondrocyte P16 fluorescence intensity | 6 B-cell donor mice/group | Raw values; mean ± SD | Welch ANOVA + prespecified Welch contrasts | Two-sided | OA-24W+anti-MIF vs OA-24W+IgG | Holm adjustment across 5 prespecified comparisons | -3.7350 | -4.5848 to -2.8852 | 2.873e-04 | *** |
| Fig. 5P | Chondrocyte P16 fluorescence intensity | 6 B-cell donor mice/group | Raw values; mean ± SD | Welch ANOVA + prespecified Welch contrasts | Two-sided | Mif-/- vs OA-24W+anti-MIF | Holm adjustment across 5 prespecified comparisons | -0.0067 | -0.1639 to 0.1506 | 1.000000 | NS |
| Fig. 5P | Chondrocyte P16 fluorescence intensity | 6 B-cell donor mice/group | Raw values; mean ± SD | Welch ANOVA + prespecified Welch contrasts | Two-sided | Mif-/-+rMIF vs Mif-/- | Holm adjustment across 5 prespecified comparisons | 5.7417 | 4.8913 to 6.5921 | 4.460e-05 | **** |
| Fig. 5Q | Chondrocyte P21 fluorescence intensity | 6 B-cell donor mice/group | Raw values; mean ± SD | Welch ANOVA + prespecified Welch contrasts | Two-sided | OA-24W+IgG vs Control | Holm adjustment across 5 prespecified comparisons | 5.4200 | 4.3572 to 6.4828 | 1.507e-04 | *** |
| Fig. 5Q | Chondrocyte P21 fluorescence intensity | 6 B-cell donor mice/group | Raw values; mean ± SD | Welch ANOVA + prespecified Welch contrasts | Two-sided | OA-24W+anti-MIF vs Control | Holm adjustment across 5 prespecified comparisons | 0.1200 | -0.0708 to 0.3108 | 0.381848 | NS |
| Fig. 5Q | Chondrocyte P21 fluorescence intensity | 6 B-cell donor mice/group | Raw values; mean ± SD | Welch ANOVA + prespecified Welch contrasts | Two-sided | OA-24W+anti-MIF vs OA-24W+IgG | Holm adjustment across 5 prespecified comparisons | -5.3000 | -6.3625 to -4.2375 | 1.507e-04 | *** |
| Fig. 5Q | Chondrocyte P21 fluorescence intensity | 6 B-cell donor mice/group | Raw values; mean ± SD | Welch ANOVA + prespecified Welch contrasts | Two-sided | Mif-/- vs OA-24W+anti-MIF | Holm adjustment across 5 prespecified comparisons | -0.0300 | -0.2022 to 0.1422 | 0.699813 | NS |
| Fig. 5Q | Chondrocyte P21 fluorescence intensity | 6 B-cell donor mice/group | Raw values; mean ± SD | Welch ANOVA + prespecified Welch contrasts | Two-sided | Mif-/-+rMIF vs Mif-/- | Holm adjustment across 5 prespecified comparisons | 7.0600 | 5.7845 to 8.3355 | 1.436e-04 | *** |
| Fig. 5R | SA-beta-gal-positive chondrocytes (%) | 6 B-cell donor mice/group | Raw values; mean ± SD | Welch ANOVA + prespecified Welch contrasts | Two-sided | OA-24W+IgG vs Control | Holm adjustment across 5 prespecified comparisons | 35.6283 | 26.7497 to 44.5070 | 5.751e-04 | *** |
| Fig. 5R | SA-beta-gal-positive chondrocytes (%) | 6 B-cell donor mice/group | Raw values; mean ± SD | Welch ANOVA + prespecified Welch contrasts | Two-sided | OA-24W+anti-MIF vs Control | Holm adjustment across 5 prespecified comparisons | 0.4767 | -0.2309 to 1.1842 | 0.323861 | NS |
| Fig. 5R | SA-beta-gal-positive chondrocytes (%) | 6 B-cell donor mice/group | Raw values; mean ± SD | Welch ANOVA + prespecified Welch contrasts | Two-sided | OA-24W+anti-MIF vs OA-24W+IgG | Holm adjustment across 5 prespecified comparisons | -35.1517 | -44.0281 to -26.2752 | 5.751e-04 | *** |
| Fig. 5R | SA-beta-gal-positive chondrocytes (%) | 6 B-cell donor mice/group | Raw values; mean ± SD | Welch ANOVA + prespecified Welch contrasts | Two-sided | Mif-/- vs OA-24W+anti-MIF | Holm adjustment across 5 prespecified comparisons | -0.1000 | -1.3158 to 1.1158 | 0.853809 | NS |
| Fig. 5R | SA-beta-gal-positive chondrocytes (%) | 6 B-cell donor mice/group | Raw values; mean ± SD | Welch ANOVA + prespecified Welch contrasts | Two-sided | Mif-/-+rMIF vs Mif-/- | Holm adjustment across 5 prespecified comparisons | 56.5017 | 47.7462 to 65.2571 | 5.739e-05 | **** |
| Fig. 6E | Mean microsphere diameter (µm) | 12 independent batches/formulation | Raw batch-level values; mean ± SD | Unpaired Student's t test | Two-sided | siMIF PLGA vs Empty PLGA | Not applicable; single prespecified comparison | 0.6500 µm | -0.1040 to 1.4040 | 0.087570 | NS |
| Fig. 6G | MIF fluorescence intensity | 6 chondrocyte donor mice/group | Raw values; mean ± SD | Welch ANOVA + Games-Howell | Two-sided | Saline vs Cy3-empty µs | Games-Howell adjustment across 3 pairwise comparisons | 0.0460 | -0.1794 to 0.2714 | 0.804249 | NS |
| Fig. 6G | MIF fluorescence intensity | 6 chondrocyte donor mice/group | Raw values; mean ± SD | Welch ANOVA + Games-Howell | Two-sided | Saline vs Cy3-siMIF µs | Games-Howell adjustment across 3 pairwise comparisons | 0.7578 | 0.5329 to 0.9827 | 9.528 × 10^-5 | **** |
| Fig. 6G | MIF fluorescence intensity | 6 chondrocyte donor mice/group | Raw values; mean ± SD | Welch ANOVA + Games-Howell | Two-sided | Cy3-empty µs vs Cy3-siMIF µs | Games-Howell adjustment across 3 pairwise comparisons | 0.7118 | 0.6271 to 0.7966 | 1.195 × 10^-8 | **** |
| Fig. 6K | MIF fluorescence intensity | 6 chondrocyte donor mice/group | Raw values; mean ± SD | One-way ANOVA + Tukey HSD | Two-sided | Quercetin hydrogel vs siMIF µs hydrogel | Tukey HSD adjustment across 3 pairwise comparisons | 0.5523 | 0.4011 to 0.7035 | 2.842 × 10^-7 | **** |
| Fig. 6K | MIF fluorescence intensity | 6 chondrocyte donor mice/group | Raw values; mean ± SD | One-way ANOVA + Tukey HSD | Two-sided | Quercetin hydrogel vs SQH | Tukey HSD adjustment across 3 pairwise comparisons | 0.7940 | 0.6428 to 0.9452 | 2.124 × 10^-9 | **** |
| Fig. 6K | MIF fluorescence intensity | 6 chondrocyte donor mice/group | Raw values; mean ± SD | One-way ANOVA + Tukey HSD | Two-sided | siMIF µs hydrogel vs SQH | Tukey HSD adjustment across 3 pairwise comparisons | 0.2417 | 0.0905 to 0.3929 | 0.002312 | ** |
| Fig. 7C | MIF fluorescence intensity | 6 chondrocyte donor mice/group | Raw values; mean ± SD | Welch ANOVA + prespecified Welch contrasts | Two-sided | Control vs OA | Holm adjustment across 7 prespecified comparisons | -2.9640 | -3.8062 to -2.1218 | 0.001378 | ** |
| Fig. 7C | MIF fluorescence intensity | 6 chondrocyte donor mice/group | Raw values; mean ± SD | Welch ANOVA + prespecified Welch contrasts | Two-sided | OA vs OA+QH | Holm adjustment across 7 prespecified comparisons | 2.1018 | 1.2184 to 2.9853 | 0.001378 | ** |
| Fig. 7C | MIF fluorescence intensity | 6 chondrocyte donor mice/group | Raw values; mean ± SD | Welch ANOVA + prespecified Welch contrasts | Two-sided | OA vs OA+SH | Holm adjustment across 7 prespecified comparisons | 3.6967 | 2.8563 to 4.5370 | 4.820e-04 | *** |
| Fig. 7C | MIF fluorescence intensity | 6 chondrocyte donor mice/group | Raw values; mean ± SD | Welch ANOVA + prespecified Welch contrasts | Two-sided | OA vs OA+SQH | Holm adjustment across 7 prespecified comparisons | 3.8362 | 2.9941 to 4.6782 | 4.820e-04 | *** |
| Fig. 7C | MIF fluorescence intensity | 6 chondrocyte donor mice/group | Raw values; mean ± SD | Welch ANOVA + prespecified Welch contrasts | Two-sided | OA+QH vs OA+SH | Holm adjustment across 7 prespecified comparisons | 1.5948 | 1.0709 to 2.1188 | 0.001378 | ** |
| Fig. 7C | MIF fluorescence intensity | 6 chondrocyte donor mice/group | Raw values; mean ± SD | Welch ANOVA + prespecified Welch contrasts | Two-sided | OA+QH vs OA+SQH | Holm adjustment across 7 prespecified comparisons | 1.7343 | 1.2097 to 2.2590 | 0.001378 | ** |
| Fig. 7C | MIF fluorescence intensity | 6 chondrocyte donor mice/group | Raw values; mean ± SD | Welch ANOVA + prespecified Welch contrasts | Two-sided | OA+SH vs OA+SQH | Holm adjustment across 7 prespecified comparisons | 0.1395 | -0.0150 to 0.2940 | 0.068906 | NS |
| Fig. 7D | MMP13 fluorescence intensity | 6 chondrocyte donor mice/group | Raw values; mean ± SD | Welch ANOVA + prespecified Welch contrasts | Two-sided | Control vs OA | Holm adjustment across 7 prespecified comparisons | -4.1602 | -5.1446 to -3.1758 | 4.750e-04 | *** |
| Fig. 7D | MMP13 fluorescence intensity | 6 chondrocyte donor mice/group | Raw values; mean ± SD | Welch ANOVA + prespecified Welch contrasts | Two-sided | OA vs OA+QH | Holm adjustment across 7 prespecified comparisons | 3.1903 | 2.2083 to 4.1724 | 5.428e-04 | *** |
| Fig. 7D | MMP13 fluorescence intensity | 6 chondrocyte donor mice/group | Raw values; mean ± SD | Welch ANOVA + prespecified Welch contrasts | Two-sided | OA vs OA+SH | Holm adjustment across 7 prespecified comparisons | 4.1670 | 3.1839 to 5.1501 | 4.750e-04 | *** |
| Fig. 7D | MMP13 fluorescence intensity | 6 chondrocyte donor mice/group | Raw values; mean ± SD | Welch ANOVA + prespecified Welch contrasts | Two-sided | OA vs OA+SQH | Holm adjustment across 7 prespecified comparisons | 4.2470 | 3.2628 to 5.2312 | 4.750e-04 | *** |
| Fig. 7D | MMP13 fluorescence intensity | 6 chondrocyte donor mice/group | Raw values; mean ± SD | Welch ANOVA + prespecified Welch contrasts | Two-sided | OA+QH vs OA+SH | Holm adjustment across 7 prespecified comparisons | 0.9767 | 0.7278 to 1.2255 | 1.321e-04 | *** |
| Fig. 7D | MMP13 fluorescence intensity | 6 chondrocyte donor mice/group | Raw values; mean ± SD | Welch ANOVA + prespecified Welch contrasts | Two-sided | OA+QH vs OA+SQH | Holm adjustment across 7 prespecified comparisons | 1.0567 | 0.8162 to 1.2972 | 2.455e-04 | *** |
| Fig. 7D | MMP13 fluorescence intensity | 6 chondrocyte donor mice/group | Raw values; mean ± SD | Welch ANOVA + prespecified Welch contrasts | Two-sided | OA+SH vs OA+SQH | Holm adjustment across 7 prespecified comparisons | 0.0800 | -0.0600 to 0.2200 | 0.222572 | NS |
| Fig. 7E | P16 fluorescence intensity | 6 chondrocyte donor mice/group | Raw values; mean ± SD | Welch ANOVA + prespecified Welch contrasts | Two-sided | Control vs OA | Holm adjustment across 7 prespecified comparisons | -2.2802 | -2.8121 to -1.7483 | 3.601e-04 | *** |
| Fig. 7E | P16 fluorescence intensity | 6 chondrocyte donor mice/group | Raw values; mean ± SD | Welch ANOVA + prespecified Welch contrasts | Two-sided | OA vs OA+QH | Holm adjustment across 7 prespecified comparisons | 2.1533 | 1.6209 to 2.6858 | 3.287e-04 | *** |
| Fig. 7E | P16 fluorescence intensity | 6 chondrocyte donor mice/group | Raw values; mean ± SD | Welch ANOVA + prespecified Welch contrasts | Two-sided | OA vs OA+SH | Holm adjustment across 7 prespecified comparisons | 2.0168 | 1.4851 to 2.5486 | 3.601e-04 | *** |
| Fig. 7E | P16 fluorescence intensity | 6 chondrocyte donor mice/group | Raw values; mean ± SD | Welch ANOVA + prespecified Welch contrasts | Two-sided | OA vs OA+SQH | Holm adjustment across 7 prespecified comparisons | 2.5577 | 2.0262 to 3.0891 | 2.910e-04 | *** |
| Fig. 7E | P16 fluorescence intensity | 6 chondrocyte donor mice/group | Raw values; mean ± SD | Welch ANOVA + prespecified Welch contrasts | Two-sided | OA+QH vs OA+SH | Holm adjustment across 7 prespecified comparisons | -0.1365 | -0.3431 to 0.0701 | 0.171487 | NS |
| Fig. 7E | P16 fluorescence intensity | 6 chondrocyte donor mice/group | Raw values; mean ± SD | Welch ANOVA + prespecified Welch contrasts | Two-sided | OA+QH vs OA+SQH | Holm adjustment across 7 prespecified comparisons | 0.4043 | 0.2201 to 0.5886 | 0.001925 | ** |
| Fig. 7E | P16 fluorescence intensity | 6 chondrocyte donor mice/group | Raw values; mean ± SD | Welch ANOVA + prespecified Welch contrasts | Two-sided | OA+SH vs OA+SQH | Holm adjustment across 7 prespecified comparisons | 0.5408 | 0.3731 to 0.7086 | 3.287e-04 | *** |
| Fig. 7F | HMGB1 fluorescence intensity | 6 chondrocyte donor mice/group | Raw values; mean ± SD | One-way ANOVA + prespecified pooled-variance contrasts | Two-sided | Control vs OA | Holm adjustment across 7 prespecified comparisons | 1.6058 | 1.2717 to 1.9399 | 2.362e-09 | **** |
| Fig. 7F | HMGB1 fluorescence intensity | 6 chondrocyte donor mice/group | Raw values; mean ± SD | One-way ANOVA + prespecified pooled-variance contrasts | Two-sided | OA vs OA+QH | Holm adjustment across 7 prespecified comparisons | -1.4300 | -1.7641 to -1.0959 | 1.918e-08 | **** |
| Fig. 7F | HMGB1 fluorescence intensity | 6 chondrocyte donor mice/group | Raw values; mean ± SD | One-way ANOVA + prespecified pooled-variance contrasts | Two-sided | OA vs OA+SH | Holm adjustment across 7 prespecified comparisons | -0.9312 | -1.2653 to -0.5971 | 1.672e-05 | **** |
| Fig. 7F | HMGB1 fluorescence intensity | 6 chondrocyte donor mice/group | Raw values; mean ± SD | One-way ANOVA + prespecified pooled-variance contrasts | Two-sided | OA vs OA+SQH | Holm adjustment across 7 prespecified comparisons | -2.1342 | -2.4683 to -1.8001 | 6.838e-12 | **** |
| Fig. 7F | HMGB1 fluorescence intensity | 6 chondrocyte donor mice/group | Raw values; mean ± SD | One-way ANOVA + prespecified pooled-variance contrasts | Two-sided | OA+QH vs OA+SH | Holm adjustment across 7 prespecified comparisons | 0.4988 | 0.1647 to 0.8329 | 0.005040 | ** |
| Fig. 7F | HMGB1 fluorescence intensity | 6 chondrocyte donor mice/group | Raw values; mean ± SD | One-way ANOVA + prespecified pooled-variance contrasts | Two-sided | OA+QH vs OA+SQH | Holm adjustment across 7 prespecified comparisons | -0.7042 | -1.0383 to -0.3701 | 4.116e-04 | *** |
| Fig. 7F | HMGB1 fluorescence intensity | 6 chondrocyte donor mice/group | Raw values; mean ± SD | One-way ANOVA + prespecified pooled-variance contrasts | Two-sided | OA+SH vs OA+SQH | Holm adjustment across 7 prespecified comparisons | -1.2030 | -1.5371 to -0.8689 | 3.642e-07 | **** |
| Fig. 7I | Terminal OARSI histological score | 6 mice/group | Individual mouse scores; Mean ± SD | Jeffreys-prior penalized cumulative-link ordinal logistic model | Two-sided | Sham vs OA | Holm adjustment across 6 prespecified pairwise contrasts | Proportional-odds ratio = 6.751 × 10^-5 | 6.489 × 10^-7 to 0.007024 | 0.000304 | *** |
| Fig. 7I | Terminal OARSI histological score | 6 mice/group | Individual mouse scores; Mean ± SD | Jeffreys-prior penalized cumulative-link ordinal logistic model | Two-sided | Sham vs OA+gel only | Holm adjustment across 6 prespecified pairwise contrasts | Proportional-odds ratio = 0.01193 | 0.000833 to 0.170908 | 0.004445 | ** |
| Fig. 7I | Terminal OARSI histological score | 6 mice/group | Individual mouse scores; Mean ± SD | Jeffreys-prior penalized cumulative-link ordinal logistic model | Two-sided | Sham vs OA+SQH hydrogel | Holm adjustment across 6 prespecified pairwise contrasts | Proportional-odds ratio = 0.13925 | 0.01661 to 1.16777 | 0.069210 | NS |
| Fig. 7I | Terminal OARSI histological score | 6 mice/group | Individual mouse scores; Mean ± SD | Jeffreys-prior penalized cumulative-link ordinal logistic model | Two-sided | OA vs OA+gel only | Holm adjustment across 6 prespecified pairwise contrasts | Proportional-odds ratio = 176.717 | 3.2156 to 9711.717 | 0.034083 | * |
| Fig. 7I | Terminal OARSI histological score | 6 mice/group | Individual mouse scores; Mean ± SD | Jeffreys-prior penalized cumulative-link ordinal logistic model | Two-sided | OA vs OA+SQH hydrogel | Holm adjustment across 6 prespecified pairwise contrasts | Proportional-odds ratio = 2062.628 | 25.0414 to 169896.200 | 0.003482 | ** |
| Fig. 7I | Terminal OARSI histological score | 6 mice/group | Individual mouse scores; Mean ± SD | Jeffreys-prior penalized cumulative-link ordinal logistic model | Two-sided | OA+gel only vs OA+SQH hydrogel | Holm adjustment across 6 prespecified pairwise contrasts | Proportional-odds ratio = 11.6719 | 1.4372 to 94.7897 | 0.042967 | * |
| Fig. 7J | MMP13 fluorescence intensity | 6 mice/group | Raw values; mean ± SD | Welch ANOVA + prespecified Welch contrasts | Two-sided | Sham vs OA | Holm adjustment across 6 prespecified comparisons | -1.2148 | -1.6891 to -0.7405 | 0.004080 | ** |
| Fig. 7J | MMP13 fluorescence intensity | 6 mice/group | Raw values; mean ± SD | Welch ANOVA + prespecified Welch contrasts | Two-sided | Sham vs OA+gel only | Holm adjustment across 6 prespecified comparisons | -0.7467 | -0.9446 to -0.5488 | 0.000262 | *** |
| Fig. 7J | MMP13 fluorescence intensity | 6 mice/group | Raw values; mean ± SD | Welch ANOVA + prespecified Welch contrasts | Two-sided | Sham vs OA+SQH hydrogel | Holm adjustment across 6 prespecified comparisons | −0.1232 | -0.2588 to 0.0124 | 0.107343 | NS |
| Fig. 7J | MMP13 fluorescence intensity | 6 mice/group | Raw values; mean ± SD | Welch ANOVA + prespecified Welch contrasts | Two-sided | OA vs OA+gel only | Holm adjustment across 6 prespecified comparisons | 0.4682 | -0.0099 to 0.9462 | 0.107343 | NS |
| Fig. 7J | MMP13 fluorescence intensity | 6 mice/group | Raw values; mean ± SD | Welch ANOVA + prespecified Welch contrasts | Two-sided | OA vs OA+SQH hydrogel | Holm adjustment across 6 prespecified comparisons | 1.0917 | 0.6175 to 1.5659 | 0.004499 | ** |
| Fig. 7J | MMP13 fluorescence intensity | 6 mice/group | Raw values; mean ± SD | Welch ANOVA + prespecified Welch contrasts | Two-sided | OA+gel only vs OA+SQH hydrogel | Holm adjustment across 6 prespecified comparisons | 0.6235 | 0.4174 to 0.8296 | 0.000462 | *** |
| Fig. 7J | HMGB1 fluorescence intensity | 6 mice/group | Raw values; mean ± SD | One-way ANOVA + prespecified pooled-variance contrasts | Two-sided | Sham vs OA | Holm adjustment across 6 prespecified comparisons | 1.4400 | 1.2265 to 1.6535 | 4.660 × 10^-11 | **** |
| Fig. 7J | HMGB1 fluorescence intensity | 6 mice/group | Raw values; mean ± SD | One-way ANOVA + prespecified pooled-variance contrasts | Two-sided | Sham vs OA+gel only | Holm adjustment across 6 prespecified comparisons | 0.6928 | 0.4794 to 0.9063 | 4.158 × 10^-6 | **** |
| Fig. 7J | HMGB1 fluorescence intensity | 6 mice/group | Raw values; mean ± SD | One-way ANOVA + prespecified pooled-variance contrasts | Two-sided | Sham vs OA+SQH hydrogel | Holm adjustment across 6 prespecified comparisons | 0.2120 | -0.0015 to 0.4255 | 0.051441 | NS |
| Fig. 7J | HMGB1 fluorescence intensity | 6 mice/group | Raw values; mean ± SD | One-way ANOVA + prespecified pooled-variance contrasts | Two-sided | OA vs OA+gel only | Holm adjustment across 6 prespecified comparisons | -0.7472 | -0.9606 to -0.5337 | 1.856 × 10^-6 | **** |
| Fig. 7J | HMGB1 fluorescence intensity | 6 mice/group | Raw values; mean ± SD | One-way ANOVA + prespecified pooled-variance contrasts | Two-sided | OA vs OA+SQH hydrogel | Holm adjustment across 6 prespecified comparisons | -1.2280 | -1.4415 to -1.0145 | 6.795 × 10^-10 | **** |
| Fig. 7J | HMGB1 fluorescence intensity | 6 mice/group | Raw values; mean ± SD | One-way ANOVA + prespecified pooled-variance contrasts | Two-sided | OA+gel only vs OA+SQH hydrogel | Holm adjustment across 6 prespecified comparisons | -0.4808 | -0.6943 to -0.2674 | 0.000276 | *** |
| Fig. 7L | MIF fluorescence intensity | 6 mice/group | Raw values; mean ± SD | One-way ANOVA + prespecified pooled-variance contrasts | Two-sided | Sham vs OA | Holm adjustment across 6 prespecified comparisons | -1.3827 | -1.6864 to -1.0789 | 3.757 × 10^-8 | **** |
| Fig. 7L | MIF fluorescence intensity | 6 mice/group | Raw values; mean ± SD | One-way ANOVA + prespecified pooled-variance contrasts | Two-sided | Sham vs OA+gel only | Holm adjustment across 6 prespecified comparisons | -0.7722 | -1.0759 to -0.4684 | 0.000103 | *** |
| Fig. 7L | MIF fluorescence intensity | 6 mice/group | Raw values; mean ± SD | One-way ANOVA + prespecified pooled-variance contrasts | Two-sided | Sham vs OA+SQH hydrogel | Holm adjustment across 6 prespecified comparisons | 0.1040 | -0.1998 to 0.4078 | 0.483391 | NS |
| Fig. 7L | MIF fluorescence intensity | 6 mice/group | Raw values; mean ± SD | One-way ANOVA + prespecified pooled-variance contrasts | Two-sided | OA vs OA+gel only | Holm adjustment across 6 prespecified comparisons | 0.6105 | 0.3067 to 0.9143 | 0.000898 | *** |
| Fig. 7L | MIF fluorescence intensity | 6 mice/group | Raw values; mean ± SD | One-way ANOVA + prespecified pooled-variance contrasts | Two-sided | OA vs OA+SQH hydrogel | Holm adjustment across 6 prespecified comparisons | 1.4867 | 1.1829 to 1.7904 | 1.340 × 10^-8 | **** |
| Fig. 7L | MIF fluorescence intensity | 6 mice/group | Raw values; mean ± SD | One-way ANOVA + prespecified pooled-variance contrasts | Two-sided | OA+gel only vs OA+SQH hydrogel | Holm adjustment across 6 prespecified comparisons | 0.8762 | 0.5724 to 1.1799 | 2.795 × 10^-5 | **** |
| Fig. 7M | HMGB1-positive B cells (%) | 6 mice/group | Raw values; mean ± SD | One-way ANOVA + prespecified pooled-variance contrasts | Two-sided | Sham vs OA | Holm adjustment across 6 prespecified comparisons | 18.1528 | 14.5384 to 21.7673 | 8.632 × 10^-9 | **** |
| Fig. 7M | HMGB1-positive B cells (%) | 6 mice/group | Raw values; mean ± SD | One-way ANOVA + prespecified pooled-variance contrasts | Two-sided | Sham vs OA+gel only | Holm adjustment across 6 prespecified comparisons | 12.6598 | 9.0454 to 16.2743 | 1.839 × 10^-6 | **** |
| Fig. 7M | HMGB1-positive B cells (%) | 6 mice/group | Raw values; mean ± SD | One-way ANOVA + prespecified pooled-variance contrasts | Two-sided | Sham vs OA+SQH hydrogel | Holm adjustment across 6 prespecified comparisons | 4.6598 | 1.0454 to 8.2743 | 0.014106 | * |
| Fig. 7M | HMGB1-positive B cells (%) | 6 mice/group | Raw values; mean ± SD | One-way ANOVA + prespecified pooled-variance contrasts | Two-sided | OA vs OA+gel only | Holm adjustment across 6 prespecified comparisons | -5.4930 | -9.1075 to -1.8785 | 0.009627 | ** |
| Fig. 7M | HMGB1-positive B cells (%) | 6 mice/group | Raw values; mean ± SD | One-way ANOVA + prespecified pooled-variance contrasts | Two-sided | OA vs OA+SQH hydrogel | Holm adjustment across 6 prespecified comparisons | -13.4930 | -17.1075 to -9.8785 | 8.821 × 10^-7 | **** |
| Fig. 7M | HMGB1-positive B cells (%) | 6 mice/group | Raw values; mean ± SD | One-way ANOVA + prespecified pooled-variance contrasts | Two-sided | OA+gel only vs OA+SQH hydrogel | Holm adjustment across 6 prespecified comparisons | -8.0000 | -11.6145 to -4.3855 | 0.000500 | *** |
